# Long-term persistence and function of hematopoietic stem cell-derived chimeric antigen receptor T cells in a nonhuman primate model of HIV/AIDS

**DOI:** 10.1371/journal.ppat.1006753

**Published:** 2017-12-28

**Authors:** Anjie Zhen, Christopher W. Peterson, Mayra A. Carrillo, Sowmya Somashekar Reddy, Cindy S. Youn, Brianna B. Lam, Nelson Y. Chang, Heather A. Martin, Jonathan W. Rick, Jennifer Kim, Nick C. Neel, Valerie K. Rezek, Masakazu Kamata, Irvin S. Y. Chen, Jerome A. Zack, Hans-Peter Kiem, Scott G. Kitchen

**Affiliations:** 1 Department of Medicine, Division of Hematology and Oncology, David Geffen School of Medicine at University of California, Los Angeles, Los Angeles, California, United States of America; 2 Stem Cell and Gene Therapy Program, Fred Hutchinson Cancer Research Center, Seattle, Washington, United States of America; 3 Departments of Medicine, University of Washington, Seattle, Washington, United States of America; 4 Department of Microbiology, Immunology and Molecular Genetics, David Geffen School of Medicine at University of California, Los Angeles, California, United States of America; 5 Department of Pathology, University of Washington, Seattle, Washington, United States of America; Miller School of Medicine, UNITED STATES

## Abstract

Chimeric Antigen Receptor (CAR) T-cells have emerged as a powerful immunotherapy for various forms of cancer and show promise in treating HIV-1 infection. However, significant limitations are persistence and whether peripheral T cell-based products can respond to malignant or infected cells that may reappear months or years after treatment remains unclear. Hematopoietic Stem/Progenitor Cells (HSPCs) are capable of long-term engraftment and have the potential to overcome these limitations. Here, we report the use of a protective CD4 chimeric antigen receptor (C46CD4CAR) to redirect HSPC-derived T-cells against simian/human immunodeficiency virus (SHIV) infection in pigtail macaques. CAR-containing cells persisted for more than 2 years without any measurable toxicity and were capable of multilineage engraftment. Combination antiretroviral therapy (cART) treatment followed by cART withdrawal resulted in lower viral rebound in CAR animals relative to controls, and demonstrated an immune memory-like response. We found CAR-expressing cells in multiple lymphoid tissues, decreased tissue-associated SHIV RNA levels, and substantially higher CD4/CD8 ratios in the gut as compared to controls. These results show that HSPC-derived CAR T-cells are capable of long-term engraftment and immune surveillance. This study demonstrates for the first time the safety and feasibility of HSPC-based CAR therapy in a large animal preclinical model.

## Author summary

Hematopoietic Stem/Progenitor Cell (HSPC) based gene therapy can be used to treat many infectious and genetic diseases. Here, we used an HSPC-based approach to redirect and enhance host immunity against HIV-1. We engineered HSPCs to carry chimeric antigen receptor (CAR) genes that detect and destroy HIV-infected cells. CAR therapy has shown huge potential in the treatment of cancer, but has only been applied in peripheral blood T-cells. HSPC-based CAR therapy has several benefits over T cell gene therapy, as it allows for normal T cell development, selection, and persistence of the engineered cells for the lifetime of the patient. We used a CAR molecule that hijacks the essential interaction between the virus and the cell surface molecule CD4 to redirect HSPC-derived T-cells against infected cells. We observed >2 years of stable production of CAR-expressing cells without any adverse events, and wide distribution of these cells in lymphoid tissues and gastrointestinal tract, which are major anatomic sites for HIV replication and persistence in suppressed patients. Most importantly, HSPC-derived CAR T-cells functionally responded to infected cells. This study demonstrates for the first time the safety and feasibility of HSPC based therapy utilizing an HIV-specific CAR for suppressed HIV infection.

## Introduction

HIV-1 specific cytotoxic T lymphocytes mount a key immune response to HIV and are crucial for the control of viremia and the elimination of HIV infected cells. Previous studies have shown that a chimeric antigen receptor containing the CD4 molecule linked to the signaling domain of the T cell receptor ζ chain (CD4CAR) can be used to redirect peripheral T cells to target HIV infected cells[1]. CD4 CAR modified T cells can recognize and respond to HIV gp120 envelope protein on infected cells and can effectively kill HIV infected cells and limit HIV replication *in vitro*. Early clinical trials with CD4CAR modified T cells were shown to be safe but had limited antiviral efficacy[2,3]. The lack of *in vivo* functionality of the transferred T cells may have been due to suboptimal T-cell handling and expansion, or because CD4CAR-expressing CD8^+^ T-cells were susceptible to HIV infection and elimination[1,2]. Protection of the CD4CAR modified cells from viral entry is essential in order to ensure T-cell functionality and survival[4,5].

Hematopoietic Stem/progenitor Cell (HSPC) -based gene therapy has several advantages over T cell adoptive therapy. First, the regenerative nature of HSPC provides a lifelong supply of engineered T cells against antigen-expressing target cells, which is key to achieve long term immune surveillance and a functional cure of HIV infection. Secondly, modified cells undergo normal T cell differentiation and selection, eliminating potentially self-reactive T cells and increasing the potential for the development of immunological memory[6–8]. Our previous studies using humanized mice demonstrated that HSPCs modified with a protective CD4CAR resulted in successful differentiation of CD4CAR expressing T cells and significant suppression of HIV replication, suggesting a high degree of feasibility in redirecting immunity with an HSPC-based approach[4].

The nonhuman primate model is an ideal preclinical surrogate for the development of cure strategies in HIV^+^ patients. A series of well-characterized HIV-like viruses are available to recapitulate acute, chronic, and cART-suppressed infection [9,10]. Furthermore, use of a large animal model facilitates the detailed measurement of viral reservoirs in peripheral blood and in tissues. We and others have used macaque models extensively to evaluate autologous HSPC transplantation to combat a number of human diseases, including HIV infection. We have previously demonstrated that infection with the highly CCR5-tropic, HIV-enveloped simian/human immunodeficiency virus SHIV-1157ipd3N4 (“SHIV-C”) resembles suppressed infection in patients, including suppression by cART, rebound following cART withdrawal, and seeding of viral reservoirs in tissues [11,12]. Further, we have shown that autologous HSPCs can be modified to resist infection, for example via CCR5 gene editing [13], or expression of a potent inhibitor of HIV/SHIV fusion, the enfuvirtide-related peptide C46[14]. We have optimized these experiments in pigtail macaques (*M*. *nemestrina*), which carry a TRIM5 genotype that is permissive to lentivirus-mediated gene therapy approaches [15]. In short, our nonhuman primate model recapitulates virological and immune facets of HIV infection in patients, and facilitates evaluation of gene therapy-based HIV cure strategies.

Here, we asked whether HIV/SHIV-specific immunity could be engendered in HSPCs and their progeny, via modification of autologous HSPCs with a C46CD4CAR-expressing lentivirus vector. Modified cells were evaluated *in vitro*, and in SHIV-infected nonhuman primates.

## Results

### C46CD4CAR-modified cells are infection-resistant and HIV-antigen specific

**Fig 1:**
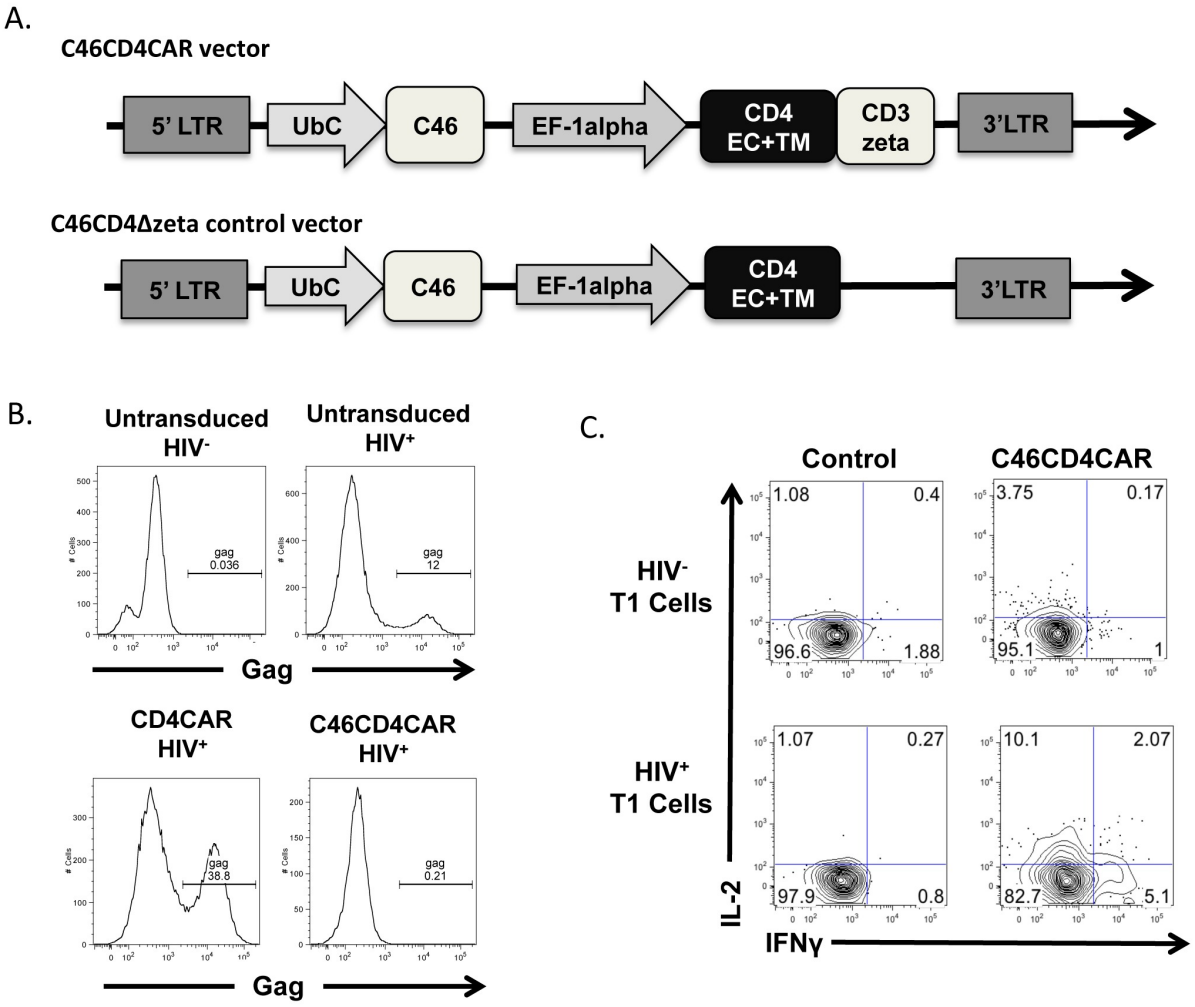
C46CD4CAR cells resist HIV infection and respond to cognate antigen. A) Lentiviral vector schematics. B) Jurkat cells were mock transduced, or transduced with CD4CAR or C46CD4CAR, then infected with HIV and cultured for 3 days. Intracellular expression of HIV-1 Gag was measured by flow cytometry using KC57 antibody. C) Pigtail macaque T cells were activated and transduced with either control or C46CD4CAR vector. 2 days following transduction, cells were stimulated with either uninfected control T1 cells or HIV-infected T1 cells. Intracellular cytokines were measured by flow analysis.

Our lentivirus constructs contain a CD4 based CAR (CD4CAR) that is composed of the human CD4 extracellular and transmembrane domain linked to the human CD3ζ signaling domain (4). CD4 is the primary receptor for HIV, hence to protect CD4CAR expressing T cells from viral infection, we co-expressed the C46 fusion inhibitor in the CAR-containing vector (C46CD4CAR)(Fig 1A) [16]. We also generated a control vector that contains C46 and a truncated form of CD4CAR that lacks the signaling domain of CD3 ζ (C46CD4CARΔzeta) (Fig 1A). Expression of CD4CAR (without C46) resulted in increased HIV infection of Jurkat T cells (35.8% HIV^+^, as compared to 12% for unmodified cells) (Fig 1B). However, expression of C46CD4CAR blocked HIV infection (0.21% HIV^+^ cells), indicating that C46 protected gene modified cells from HIV infection. We next tested if the CD4CAR molecule can functionally respond to antigen in pigtail macaque T cells. We transduced pigtail macaque T cells with control C46CD4CARΔzeta or C46CD4CAR vector, then stimulated the cells with either uninfected or HIV-infected cells that expressed HIV envelope. C46CD4CAR transduced pigtail macaque T cells produced IL-2 and IFNγ in response to stimulation, indicating that the CD4CAR molecule is functional in the NHP cells (Fig 1C). In contrast, control cells that expressed C46CD4CARΔzeta did not respond to HIV infected cells. These data show that C46CD4CAR cells are protected against HIV infection and respond functionally and specifically to HIV antigen in NHP cells *in vitro*.

### Multilineage engraftment of C46CD4CAR-modified HSPCs

**Fig 2:**
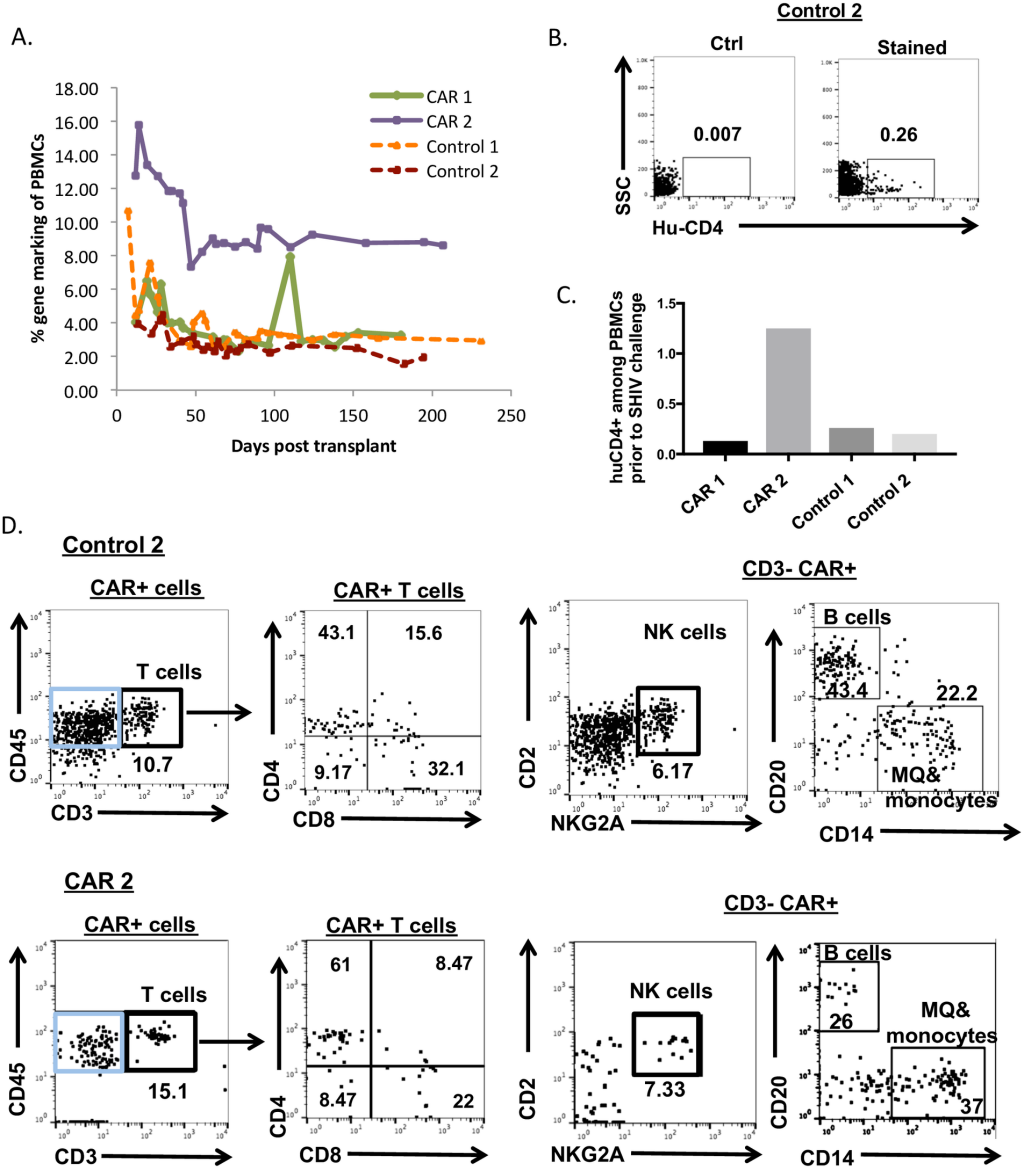
Engraftment of C46CD4CAR modified HSPCs is multilineage. A) Lentivirus gene marking in transplanted animals following autologous transplantation, measured by Taqman. B) Detection of C46CD4CAR-modified cells by flow cytometry with anti-human CD4 antibody clone 13B8.2, which does not detect Macaca nemestrina CD4. C) Percent of C46CD4CAR cells among total peripheral PBMCs, at approximately 28 days post-transplant. D) Multilineage engraftment of lentivirus-modified cells in peripheral blood was measured from control and C46CD4CAR animals. CAR+ cells were first gated on huCD4+ cells. T cells are defined as CD45+CD3+, CD3-CAR+ (blue gate) cells are gated and NK cells are defined as CD45+CD3-CD2+NKG2A+, B cells are defined as CD45+CD3-CD20+, macrophage (MQ)&monocytes are defined as CD45+CD3-CD14+. One representative control (top panels) and CAR animal (bottom panels) are shown.

To examine the effects of the CD4CAR *in vivo*, four male juvenile pigtail macaques were transplanted with autologous HSPCs that were transduced with lentivirus expressing C46CD4CAR (“CAR1” and “CAR2”) or C46CD4CARΔzeta (“Control 1” and “Control 2”). As shown in S1 Fig, percent lentivirus marking from each animal’s HSPC infusion product ranged from 4.65% to 40% in colony forming assays. After HSPC transplant, recovery kinetics of total white blood cells, platelets, neutrophils, and lymphocytes in both control and CAR animals were normal [14,17] (S2 Fig). We detected stable gene marking of PBMCs from all animals prior to SHIV challenge (Fig 2A). In addition, we were able to detect C46CD4CAR or C46CD4CARΔzeta modified cells in peripheral blood by using an anti-human CD4 antibody clone (13B8.2) that detects human, but not pigtail macaque CD4 (Fig 2B). Because this antibody will only label the human CD4CAR in our animals, we refer to CAR^+^ cells as “huCD4^+^”. We found that 0.1% to 1.25% of CD45^+^ peripheral blood leukocytes from control or CAR animals were huCD4^+^ (Fig 2C). Importantly, huCD4^+^ CAR cells from both control and CD4CAR animals differentiated into multiple hematopoietic lineages, including T cells (CD45^+^CD3^+^), NK cells (CD3^−^CD2^+^NKG2A^+^)[18], B cells (CD45^+^CD3^−^CD20^+^) and monocytes and macrophages (CD3^−^CD20^−^CD14^+^) (Fig 2D). These results show that autologous transplantation of C46CD4CAR-transduced HSPC is safe and well tolerated, and results in stable, multilineage engraftment with typical kinetics of hematopoietic recovery.

### Reduced SHIV rebound in C46CD4CAR animals

**Fig 3:**
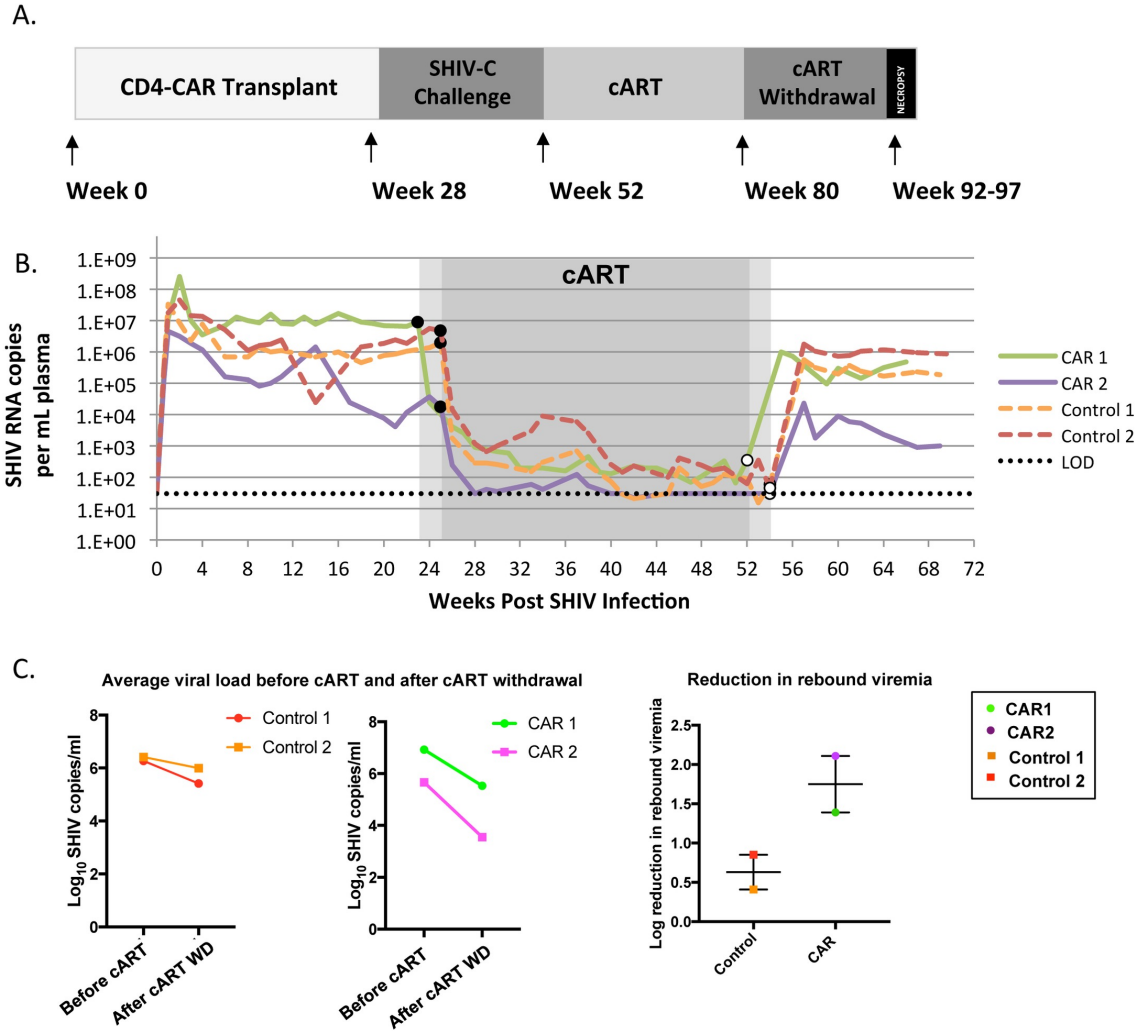
Reduced viral rebound in C46CD4CAR animals. A) CD4-CAR and Δzeta control animals were challenged with SHIV-1157ipd3N4 (“SHIV-C”) via the intravenous route. Approximately 24 weeks later, cART was initiated and administered for 28 weeks. Following cART withdrawal, animals were monitored for approximately 15 weeks prior to necropsy. B) Plasma viral load was monitored longitudinally after SHIV challenge. Dashed lines indicate limit of detection (LOD) of 30 copies/mL. Closed circles and open circles indicate beginning and end of cART, respectively. C) Average viral load before cART and after cART withdrawal, and log reduction of plasma viremia following post-cART viral rebound, relative to comparable time points during primary infection. Average viral load calculations are described in Materials and Methods.

To study the effect of C46CD4CAR transplantation on SHIV replication, animals were infected with SHIV-C for ~24 weeks followed by 28 weeks of combination antiretroviral therapy (cART) and subsequent cART withdrawal. At least 12 weeks after cART cessation, animals were then sacrificed for necropsy (Fig 3A). Both CD4CAR and control animals had slightly higher plasma viral loads prior to cART, and did not achieve full virus suppression following cART (Fig 3B). This was likely due to residual immune suppression from the transplant procedure [11,19]. The CAR1 animal had approximately 1 log higher viral load as compared to control animals during acute and chronic SHIV infection, while the CAR2 animal, in which more than 1% of PBMCs were modified with C46CD4CAR prior to SHIV infection, had lower peak viremia during acute infection and showed progressively decreasing viral loads prior to cART (Fig 3B). While CAR 1 and control animals appear to have reached set points after 4 weeks of SHIV infection, CAR 2 animal demonstrated a trend of continuous reduction of viral load throughout primary infection (Fig 3B). Interestingly, when we compared average viral load after cART withdrawal to average viral load during primary infection (week 2 to week 22), we found that both CAR containing animals had lower average rebound viremia (1.4–2.11 log lower than primary setpoint) as compared to the control animals (0.4–0.8 log lower than primary setpoint) (Fig 3C). These findings are consistent with a model in which C46CD4CAR cells are capable of establishing virus-specific immune memory and responding to recrudescent SHIV viremia.

### C46CD4CAR cells expand in a SHIV-dependent manner in vivo

**Fig 4:**
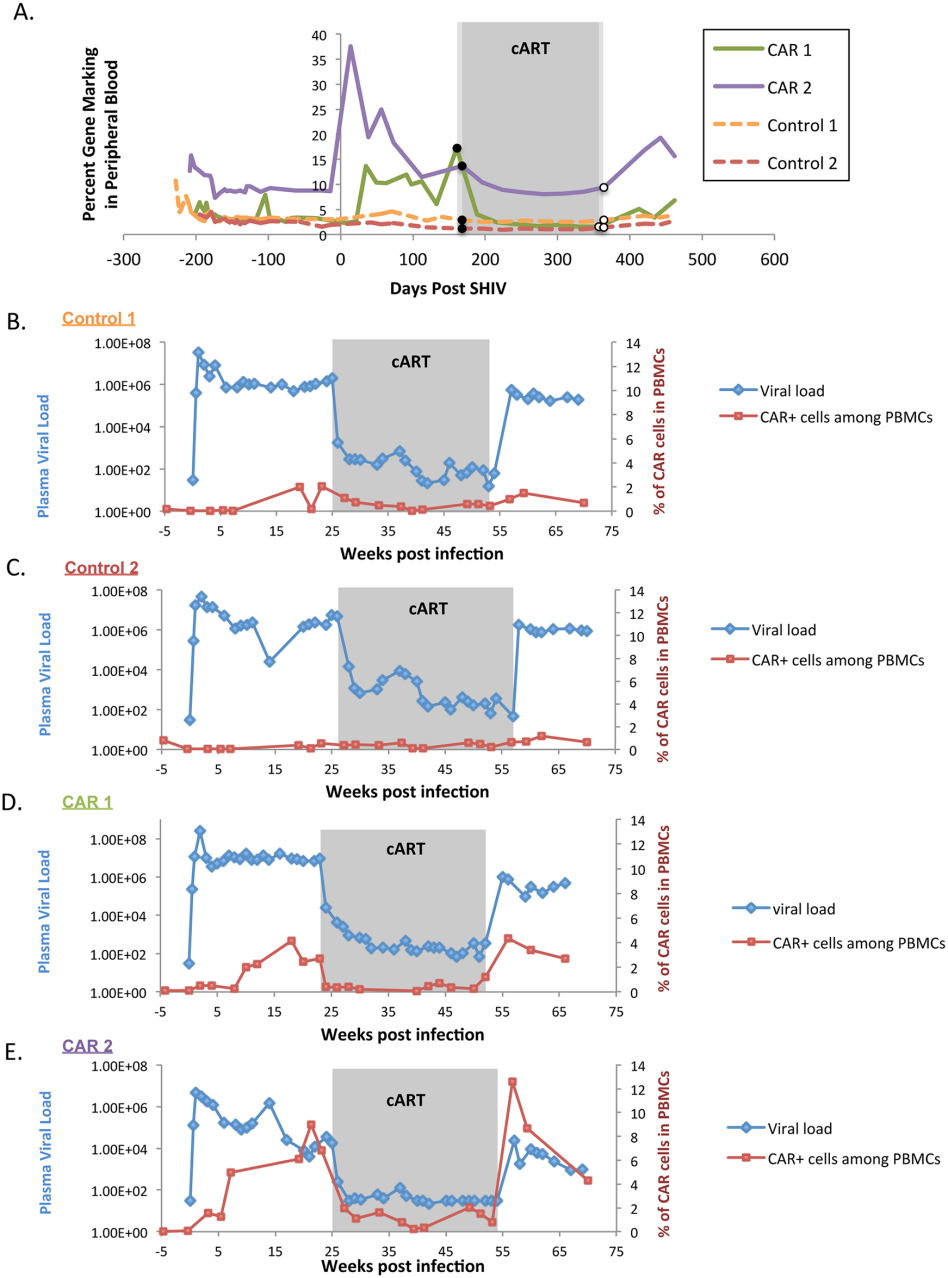
C46CD4CAR cells expand in response to SHIV antigen in vivo. A) Lentiviral gene marking was measured by Taqman from peripheral blood at the indicated time points from C46CD4CAR (solid lines) and C46CD4CAR Δzeta transplanted animals (dashed lines). (B-E) Viral load (left Y axis, blue) and percentage of CAR cells in PBMCs (right Y axis, red) were measured longitudinally from peripheral blood from Control1 (B), Control 2 (C), CAR 1 (D), and CAR 2 (E) animals.

We next measured antigen-dependent responses in C46CD4CAR and control animals by monitoring CAR gene marking as a function of SHIV plasma viremia. Lentivirus-marked cells were readily detectable by Taqman in CAR and control animals over the course of our nearly two-year study (Fig 4A). Interestingly, CAR animals, but not controls, showed increased gene marking in the periphery at multiple time points. These were coincident with increases in SHIV viremia, notably during primary infection and viral rebound after cART withdrawal (Fig 3B). To investigate further, we used flow cytometry to stain for huCD4^+^ PBMCs at multiple time points following SHIV infection. Consistent with Taqman-based gene marking data, we found that huCD4^+^ cells from CAR animals, but not control animals, expanded upon SHIV infection and post-cART withdrawal viral rebound (Fig 4B–4E). This confirms that functional C46CD4CAR cells require intact CD3ζ signaling in order to expand in response to SHIV antigen. Furthermore, we observed an increase of C46CD4CAR^+^ cells during acute and chronic SHIV infection (Fig 4D and 4E), reminiscent of a primary immune response to infection. After the cessation of cART treatment, the percentage of CD4CAR^+^ cells again increased rapidly, mimicking a memory response. The CAR2 animal, which had higher gene marking prior to SHIV infection (Fig 2A), contained as many as 10% and 12.6% huCD4^+^ PBMCs during primary untreated infection and after cART withdrawal, respectively. We also observed expansion in percentage of huCD4^+^ cells among T cells (S3A Fig) and in huCD4^+^ T cell numbers (S3B Fig). The expansion of CD4CAR^+^ cells is primarily driven by CAR expressing T cells as shown in Sup Fig 3A and 3B. During viral rebound, the 4–10 fold higher levels of CAR marking in C46CD4CAR animals relative to controls was consistent with the 1.5–2 log decrease in rebound viremia relative to primary infection in these animals (Fig 3C). These data suggest that CAR-marked cells engraft long term and are capable of antigen-specific expansion months or years after transplantation.

### SHIV-dependent effector differentiation and function of CD4CAR cells

**Fig 5:**
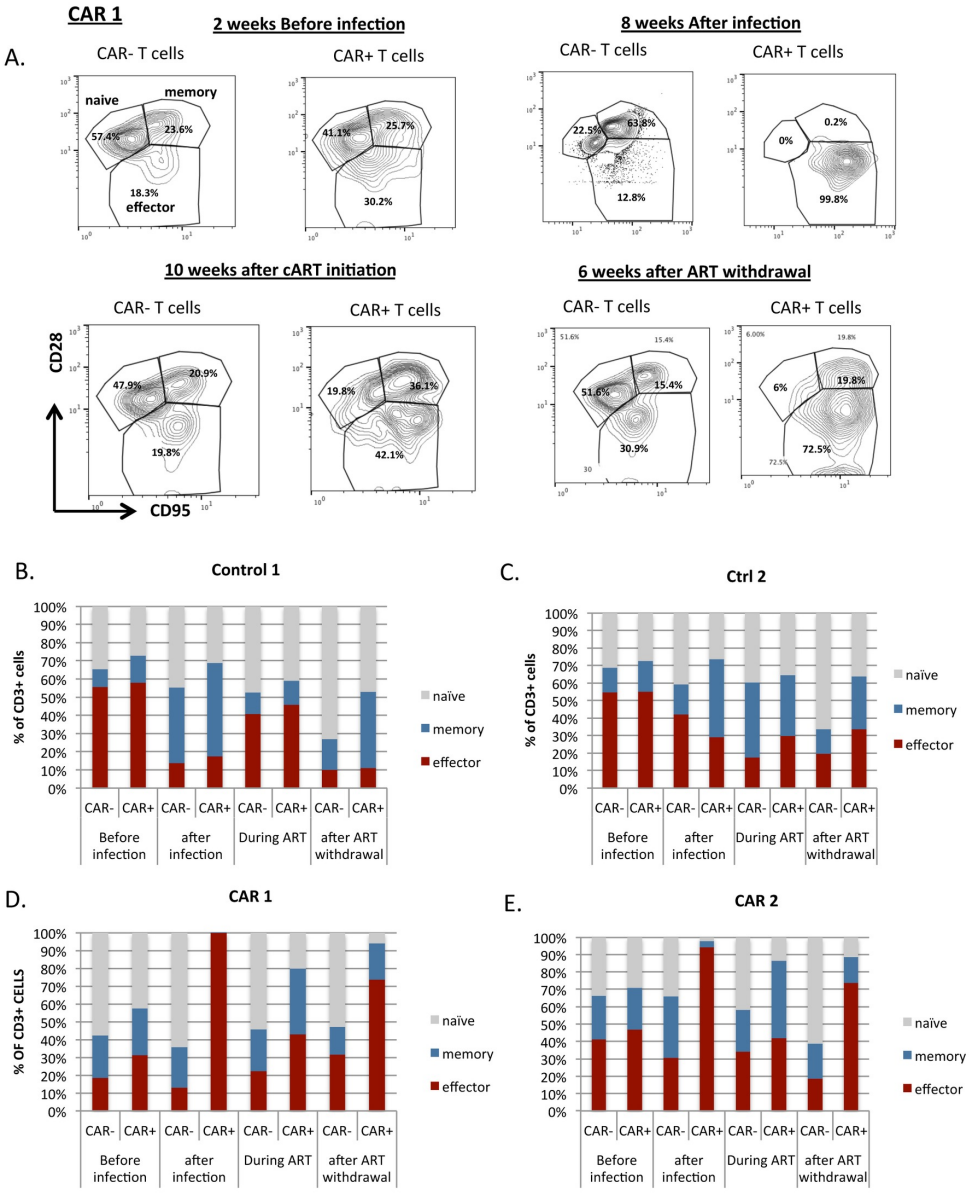
C46CD4CAR T cells develop into effector cells in response to viral replication. A) Percentages of unmarked (CAR-) naïve, memory, and effector T cells and C46CD4CAR+ T cells from peripheral blood from one representative animal, collected at the indicated time points. (B-E) Quantitation of naïve, memory, and effector subsets from unmarked T cells and gene modified T cells in peripheral blood from Control 1 (B), Control 2 (C), CAR 1 (D) and CAR 2 (E) animals.

Our *in vitro* data suggest that expansion of C46CD4CAR cells is specific to HIV/SHIV antigen (Fig 1). To examine how CAR modified cells respond to SHIV replication *in vivo*, we monitored the naïve, effector, and memory phenotypes of CAR T cells longitudinally in our transplanted animals following SHIV infection. Prior to SHIV infection, huCD4^+^ (CAR+) and unmarked T cells (CAR-) shared similar percentages of naïve (CD28^+^CD95^−^), effector (CD28^−^CD95^+^) and memory (CD28^+^CD95^+^) subsets (Fig 5A). Strikingly, huCD4^+^ cells became predominantly effector T cells after SHIV infection, consistent with a response to SHIV antigen. During cART-dependent viral suppression, when the percentage of huCD4^+^ T cells contracted, we found that most displayed a naïve or memory phenotype. After cART withdrawal, huCD4^+^ T cells again displayed a predominant effector phenotype. Antigen-dependent increases in the percentage of effector cells were observed in C46CD4CAR animals, but not in CD4CARΔzeta controls (Fig 5B–5E).

To investigate if CAR+ effector cells can mediate specific killing of HIV Env expressing cells, we performed ex vivo killing assays using PBMCs from CAR or control animals during primary SHIV infection prior to cART treatment and after cART withdrawal (S4A Fig). We used U1 cells stimulated to express HIV envelope as targets. Notably, this human cell line lacks rhesus MHC molecules, and therefore should only be killed following CAR-dependent recognition of the HIV envelope. While PBMCs from control animals failed to mediate specific killing of Env+ U1 cells, PBMCs from CAR animals effectively mediated specific killing of Env+ U1 cells over control U1 cells (S4B and S4C Fig). Interestingly, we observed a trend of an increase in IFNγ production of cryopreserved T cells from CAR animals in response to SHIV peptide pool stimulation, suggesting a potential synergy between CAR T cells and anti-SHIV natural T cell response (S5A–S5C Fig). However, we did not observe a clear difference in production of natural anti-SHIV or anti-HIV Env antibody (S6A and S6B Fig). These findings are consistent with our previous data (Figs 1 and 3), demonstrating that C46CD4CAR HSPC-derived cells generate a long-lived, functional response to SHIV antigen. Previous CD19 CAR T cell therapy has been associated with cytokine release syndrome, and the principle cytokines elevated in patients treated with CD19 CAR T cells were IFNγ and IL-6[20].

To monitor toxicity associated with the CD4CAR HSPCs treatment, we measured plasma cytokine levels from transplanted CAR and control animals blood collected prior to SHIV challenge, during untreated SHIV infection, during cART treatment and after cART withdrawal (S7A Fig). As shown in S7B Fig, most proinflammatory cytokines, including IFNγ, IL-1β, IL-2, IL-6, MIP1β, MIP1α, MCP1 have mostly undetectable levels or no clear difference between control and CAR animals (S7C Fig). We observed lower sCD40L levels for CAR control animals during untreated SHIV infection and after cART, which may be a result of reduced SHIV-mediated inflammation in CAR animals. Overall, we observed no toxicity associated with the CAR transplant as compared to control animals.

### Multilineage, antigen-dependent engraftment of C46CD4CAR cells in tissues

We extended our analysis of C46CD4CAR cells by examining trafficking to multiple tissue sites, including those that have been characterized as viral reservoirs[21–23]. Both C46CD4CAR and C46CD4CARΔzeta cells were found in multiple lymphoid tissues, including various lymph nodes, gut, and bone marrow (S8 Fig). Similar to PBMCs, CAR animals had higher percentage of huCD4+ cells among T cells in various tissues as compared to control animals(S8A Fig). As with huCD4^+^ PBMC, tissue-associated CAR cells were multilineage, including CD4^+^ and CD8^+^ T cells, NK cells, and macrophages/monocytes. There were no obvious differences in cell composition between C46CD4CAR and C46CD4CARΔzeta modified cells (S8B–S8E Fig).

**Fig 6:**
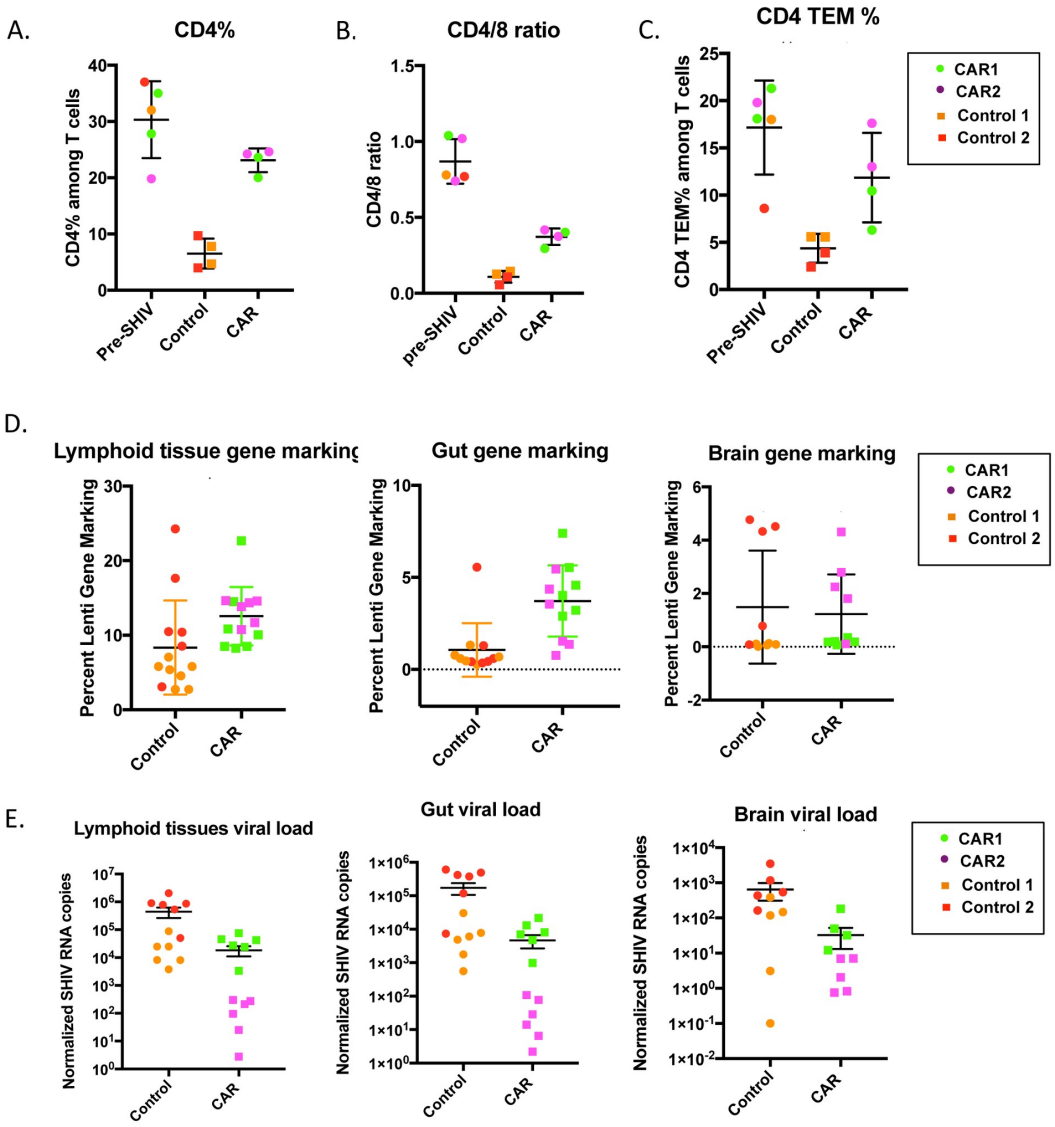
C46CD4CAR cells protect CD4^+^ T-cells and decrease viral load in multiple tissues. (A-C) GI biopsies were collected from colon and duodenum/jejunum from C46CD4CAR and control animals prior to SHIV infection, and after infection and cART withdrawal. Shown are CD4/8 ratio (A), %CD4^+^ T cells (B) and %CD4^+^ effector memory T-cells (C). D) At necropsy, Taqman was used to measure lentivirus gene marking from lymphoid tissues (spleen and mesenteric, axillary, inguinal, and submandibular lymph nodes), gastrointestinal tract (duodenum, jejunum, ileum, cecum, colon, rectum) and brain tissues (hippocampus, basal ganglia, thalamus, parietal cortex, cerebellum). E) Normalized SHIV RNA copy number from same tissues as in (D). * p<0.01, **p<0.001, ***p<0.0001 by Mann-Whitney test.

To examine the ability of C46CD4CAR cells to protect against SHIV-dependent depletion of CD4^+^ cells in the gut, biopsies were taken from the GI tract (colon or duodenum/jejunum) before SHIV infection and after cART withdrawal, and analyzed by flow cytometry. Control animals displayed a profound loss of CD4^+^ cells, both in terms of CD4^+^CD3^+^ T cell percentage (Fig 6A and S9A Fig) and CD4/8 ratio (Fig 6B and S9B Fig). Strikingly, CD4^+^ T-cell percentage and CD4/8 ratio were substantially higher in C46CD4CAR animals following cART withdrawal as compared to the control animals, suggesting that functional CAR cells contributed to protection of immune homeostasis in this compartment. Furthermore CD4^+^ effector memory T-cells (CD3^+^CD4^+^CCR7^−^CD45RA^−^), which are major target cells of HIV infection, were also protected in the gut of C46CD4CAR animals (Fig 6C and S9C Fig).

At necropsy, gene marking in lymphoid tissues (including spleen, mesenteric lymph nodes, axillary lymph nodes, inguinal lymph nodes, and submandibular lymph nodes) and gut (including duodenum, jejunum, ileum, cecum, colon, and rectum) was significantly higher in CAR animals relative to controls (Fig 6D). Interestingly, SHIV RNA measurements in these tissues showed that CAR containing animals had substantially lower viral loads (Fig 6E). Although we did not observe significant differences in gene marking in the brain (including hippocampus, basal ganglia, thalamus, parietal cortex, and cerebellum) between CAR and control animals, SHIV RNA measurements were also lower in this compartment in CAR animals, relative to controls (Fig 6D and 6E). In particular, the CAR2 animal had dramatically lower SHIV mRNA (4–5 logs) across all lymphoid tissues as compared to control animals (S10 Fig). Collectively, these results demonstrate that C46CD4CAR cells in tissues are capable of long term, multilineage engraftment, and are protected against SHIV replication, consistent with our observations in peripheral blood.

## Discussion

The seminal case study for HIV cure/remission is the Berlin patient, who received an allogeneic, HLA-matched HSPC transplant from a donor homozygous for CCR5Δ32[24], and has stimulated the search for HSPC-based cure approaches. Allogeneic HSPC transplantation without CCR5Δ32-protected donor cells in 2 HIV^+^ recipients initially resulted in undetectable HIV-1 after patients achieved full donor chimerism; this was likely due to a “graft versus reservoir” effect in which donor lymphocytes destroyed latently infected host cells. Ultimately, this intervention failed to eradicate latently infected cells, which rebounded after cART cessation[25]. These studies indicated that a combinatorial approach, rendering the blood and immune system resistant to infection and at the same time harnessing the immune system to attack infected cells, would be required. Numerous studies have used various gene therapy and gene editing approaches to genetically modify autologous stem cells, rendering them resistant to HIV infection[13,14,26–30]. Our nonhuman primate model of suppressed HIV infection is highly relevant to HIV cure studies in humans, utilizing a virus that is suppressed by a clinically relevant cART regimen, establishment of viral persistence in secondary lymphoid tissues, and rebound of viral replication following cART withdrawal[11,12]. We have previously shown that C46-expressing T-cells are protected against CCR5- and CXCR4-tropic viruses, and support a more robust immune response against infected cells *in vivo* [14,16]. Here, we demonstrate that HIV/SHIV-specific CAR cells possess strong antiviral activity even at low levels. These cells should act as sentinels, generating a robust immune response to reactivated infected cells months or years after they are introduced, without requiring expression in a high percentage of immune cells.

The most novel aspect of our approach is the generation of CAR cells from autologous HSPCs. Stem cell-based expression of CARs contributes a long-lived source of these cells, capable of providing lifelong immune surveillance against recrudescent virus. In contrast, adoptive transfer of CAR-modified T-cell products must overcome barriers including immune exhaustion, limited trafficking to tissues, and lack of functionality at these sites [31,32]. Furthermore, although CD4CAR T-cells persist long term in patients [33], it is unclear whether persisting cells are capable of responding to increased antigen loads, for example during cART treatment interruption. Here, we show for the first time in a clinically relevant large animal model, that autologous transplantation with a CAR-modified HSPC is safe and can be used to redirect long-term anti-viral immunity. We observed multilineage engraftment of autologous, gene-modified cells that persisted for almost 2 years. Moreover, we found that CAR-expressing cells expanded in response to SHIV infection in an antigen-driven fashion, and differentiated into effector cells in a CD3ζ domain-dependent manner. Intriguingly, we found that engineered CAR cells contracted during cART treatment during lower levels of antigen expression, followed by a rapid expansion after cART withdrawal, mimicking a memory response. As a result, CAR animals had decreased viremia during post-cART viral rebound, as compared to control animals. More importantly, we were able to detect CAR cells in multiple lymphoid tissues, including gut-associated lymphoid tissues. CD4^+^ T-cells at these sites facilitate viral replication, are significantly depleted during untreated infection, and are slow to regenerate during cART treatment[34]. We found significantly lowered SHIV mRNA in lymph nodes, gut and brain from C46CD4CAR animals as compared to control animals. Strikingly, both CAR animals showed substantially improved CD4/CD8 ratios and higher percentages of CD4^+^ and CD4^+^ effector memory cells in the gut after cART withdrawal, suggesting CD4^+^ T-cell protection was C46CD4CAR-dependent.

A small percentage of CAR-modified cells appeared to be sufficient to redirect an effective immune response against SHIV-infected cells in our study. For both CAR animals, we observed robust expansion of C46CD4CAR cells after SHIV infection and cART withdrawal, which was dependent on CD3ζ signaling. The immediate, memory-like response for CAR cells after cART withdrawal from both CAR animals likely contributed to improved control of SHIV viremia and CD4 protection in the gut as compared to control animals. Many of the same optimization parameters used in T-cell-based CAR products are also applicable to HSPC-based CAR cells. For example, 2^nd^ and 3^rd^ generation chimeric antigen receptors with co-stimulatory molecules such as 41BB and/or CD28 [35] may further boost the primary and secondary responses of HSPC-derived CAR T cells, although the impact of these modifications on thymopoiesis has not yet been tested. Another novel aspect of our HSPC-based approach is the generation of CAR cells in lineages other than T-cells (Figs 2 and S3). The contribution of CAR-expressing cells other than T-cells in our model remains to be determined. While CAR expressing natural killer cells can contribute to clearance of infected cells[36,37], C46CD4CAR is likely not functional in other cell types due to the lack of signaling pathway for CD3ζ[38]. If necessary, cell type-specific expression of the chimeric antigen receptor from transduced HSPCs may further improve efficacy and safety.

Our studies clearly demonstrate the potential of using CAR gene therapy in HSPCs to redirect anti-HIV immunity against HIV-1 infection. These results set the stage for future attempts to eradicate viral infection and provide more effective immune surveillance for HIV, using optimized CAR vectors and combinatorial approaches, for example with latency reversing agents and/or additive immunotherapies. Importantly, these findings have broad implications beyond HIV: additional preclinical studies should be performed to explore HSPC-expressed CARs against other infectious diseases and cancer in greater detail.

## Materials and methods

### Ethics statement

This study was carried out in strict accordance with the recommendations in the Guide for the Care and Use of Laboratory Animals of the National Institutes of Health (”The Guide”), and was approved by the Institutional Animal Care and Use Committees of the Fred Hutchinson Cancer Research Center and University of Washington, Protocol # 3235–01. All animals were housed at and included in standard monitoring procedures prescribed by the Washington National Primate Research Center (WaNPRC). This included at least twice-daily observation by animal technicians for basic husbandry parameters (e.g., food intake, activity, stool consistency, overall appearance) as well as daily observation by a veterinary technician and/or veterinarian. Animals were housed in cages approved by “The Guide” and in accordance with Animal Welfare Act regulations. Animals were fed twice daily, and were fasted for up to 14 hours prior to sedation. Environmental enrichment included grouping in compound, large activity, or run-through connected cages, perches, toys, food treats, and foraging activities. If a clinical abnormality was noted, standard WaNPRC procedures were followed to notify the veterinary staff for evaluation and determination for admission as a clinical case. Animals were sedated by administration of Ketamine HCl and/or Telazol and supportive agents prior to all procedures. Following sedation, animals were monitored according to WaNPRC standard protocols. WaNPRC surgical support staff are trained and experienced in the administration of anesthetics and have monitoring equipment available to assist, including monitors of heart rate, respiration, blood pressure, temperature, and blood oxygenation. Monitors supplied readily and easily interpretable alerts, including audible alarms, LCD readouts, etc. For minor procedures, the presence or absence of deep pain was tested by the toe-pinch reflex. The absence of response (leg flexion) to this test indicates adequate anesthesia for this procedure. Similar parameters were used in cases of general anesthesia, including the loss of palpebral reflexes (eye blink). Analgesics were provided as prescribed by the Clinical Veterinary staff for at least 48 hours after the procedures, and could be extended at the discretion of the clinical veterinarian, based on clinical signs. Decisions to euthanize animals were made in close consultation with veterinary staff, and were performed in accordance with guidelines as established by the American Veterinary Medical Association Panel on Euthanasia (2013). Prior to euthanasia, animals were first rendered unconscious by administration of ketamine HCl.

### Autologous transplantation of pigtail macaques

Four juvenile male pigtail macaques were transplanted with autologous, lentivirus modified HSPCs as previously described [14]. In short, animals were mobilized with granulocyte-colony stimulating factor (G-CSF) and stem cell factor (SCF) for 4 days prior to collection of large volume bone marrow aspirates and bead-based positive selection of CD34^+^ cells. Over a 48-hour *ex vivo* culture period, cells were transduced twice with the lentiviruses indicated in Fig 1 at a multiplicity of infection (MOI) of 5 (CAR 1) or 10 (CAR 2, Control 1, Control 2). During HSPC transduction *ex vivo*, animals received a myeloablative conditioning regimen consisting of a fractionated dose of 1020 cGy total body irradiation. Following conditioning, the HSPC product was infused back into the autologous animal. A small aliquot of the infused cell product was plated in Colony Gel Medium (Reach Bio, Seattle, WA) and analyzed as previously described[14,39]. Individual colonies and total genomic DNA (gDNA) isolated at the indicated post-transplant time points were measured by gel-based and Taqman-based PCR methods, respectively, as previously described [14,17].

### SHIV infection, cART, and peripheral blood/tissue analyses

Animals were allowed to recover for approximately 200 days prior to infection with SHIV-C, which was administered to animals via the intravenous challenge route as previously described [11]. Combination antiretroviral therapy consisted of 20 mg/kg Tenofovir and 40 mg/kg FTC dosed 1X/day subcutaneous, and 150mg Raltegravir, dosed 2X/day oral with food. Plasma viral loads, peripheral T-cell counts, longitudinal tissue surgeries, and necropsy tissue collections were conducted as previously described [12,14]. Taqman-based peripheral blood measurements were performed from gDNA isolated from total leukocytes. Total RNA and gDNA from tissue samples were isolated using a Precellys 24 homogenizer and CK28-R hard tissue homogenizing beads (Bertin Corp.) as previously described [12]. Normalized SHIV RNA copy number in tissue was calculated by normalizing SHIV RNA copy number to the crossing threshold of macaque RNase P subunit p30 RNA. PCR-based assays for SHIV were designed not to detect HIV-based lentiviral vectors and average viral loads and log reduction in viral load were calculated as described previously [12]. Briefly, average viral loads following cART withdrawal were calculated by averaging each measurement from the first detection of recrudescent plasma viremia to the final measurement taken at necropsy (11–13 weekly data points). Average viral loads before cART were calculated by averaging plasma viremia over the same number of weekly data points from primary infection, beginning with the first detection of virus following intravenous SHIV challenge.

### Flow cytometry

To detect huCD4^+^ CAR modified cells, PBMCs and tissue necropsy samples were stained with the following antibodies: anti-human specific CD4 antibody (for detection and analysis of CD4CAR modified cells; Beckman Coulter, clone 13B8.2), anti-NHP CD45 (BD Biosciences, clone D058-1283), anti-CD4 (eBiosciences, clone OKT4), anti-CD8 (eBiosciences, clone SK1), anti-CD20 (eBiosciences, clone 2H7), anti-NK2Ga (Beckman Coulter, clone A60797), anti-CD14 (Beckman Coulter, clone IM2707U), anti-CD95 (BD Biosciences, clone DX2), anti-CD28 (BD Biosciences, clone D28.2), and anti-CD3 (BD Biosciences, clone SP34-2). Fluorophore conjugates included FITC, PE, PE-Cy5, PE-Cy7, alexa700, V500, efluor450, APC, and APC-efluor780.

### Cytokine assay

To test C46CD4CAR cell functions in NHP cells, NHPPBMCs were purified from healthy pigtail macaque blood and stimulated with bead bound anti-CD3 (BD Biosciences, clone SP34-2) and anti-CD28 (BD Biosciences, clone D282.2) for 3 days. Afterwards, cells were transduced with either C46CD4CAR or C46CD4CARΔzeta lentivirus. 2 days after transduction, cells were co-incubated with either T1 cells or HIV-infected Sup-T1 cells (AIDSreagent) for 16 hours, followed by 6 hours of GolgiPlug treatment. Afterwards cells were first surface-stained with anti-CD3, anti-human CD4 (for detection of CD4CAR transduced cells) and then intracellular-stained with anti-IFNγ (eBiosciences, clone 4S B3), anti-IL-2 (BD Biosciences, clone MQ1-17H12) and analyzed by flow cytometry.

To measure natural T cell response to SHIV infection, PBMCs were isolated from transplanted pigtail macaque peripheral blood collected during primary SHIV infection prior to cART treatment, and after cART withdrawal and cryopreserved. Total PBMCs were then thawed and stimulated with either no stimulation or SIVmac Gag, Pol, tat, nef, vif and HIV Env peptide pool overnight, followed with 6 hours of GolgiPlug (BD biosciences) incubation. Afterwards, cells were harvested and stained with anti-NHP CD3, CD4, CD8, huCD4 and intracellular IFNγ.

### In vitro HIV infection

Jurkat cells (clone E6-1, AIDSreagent) were either untransduced or transduced with 2MOI CD4CAR (without C46) or C46CD4CAR for 2 days and infected with HIV-1_NL4.3_ (100 ng p24/10^6^ cells) for 3 days. Afterwards, cells were intracellularly stained with anti-Gag (clone KC57) and analyzed by flow cytometry.

### Ex vivo killing assay

PBMCs were isolated from transplanted pigtail macaque peripheral blood collected during primary SHIV infection prior to cART treatment, and after cART withdrawal. Total PBMC were co-incubated with unstimulated U1 (HIV latent cell line, Env-) or PMA activated U1 (HIV Env+) at 3:1, 10:1 and 20:1 ratios for 10 hours. In order to quantify target killing, target cells were pre-stained with celltrace Vioblue (ThermoFisher Scientific) prior to co-incubation, and percent killing was calculated as the percent loss of live celltrace Vioblue^+^ target cells after co-incubation with effector PBMC.

### Multiplex assay

Plasma was isolated from transplanted pigtail macaque peripheral blood collected during primary SHIV infection prior to cART treatment, and after cART withdrawal and frozen at -80. After necropsy, plasma was thawed and 25ul (max) was used to carry out Milliplex none-human-primate multiplex assay (EMD Millipore) for detection of proinflammatory cytokines IFNγ, IL-1β, IL-2, IL-6, MCP-1, MIP1β, MIP1α, sCD40L, TNFα. Detection range of cytokines are between 2.44 to 10,000 pg/ml. Data shown was average of 2 replicate wells.

## Supporting information

S1 Fig Lentiviral gene marking in transduced HSPC infusion products. Four male juvenile pigtail macaques were transplanted with autologous HSPCs transduced with lentiviruses expressing C46CD4CAR (CAR) or C46CD4CARΔZeta (Control). Colony forming assays were plated from a small aliquot of transduced CD34^+^ cells that were infused into each autologous recipient. “Percent Lenti+ Colonies” represents the number of lentivirus-positive colonies divided by actin-positive colonies, measured by PCR; numerical values are displayed over each bar.
(PDF)

S2 Fig Hematopoietic recovery following CD4 CAR-HSPC transplant. Four male juvenile pigtail macaques were transplanted with autologous HSPCs transduced with lentiviruses expressing either CD4 chimeric antigen receptor (C46CD4CAR, green and purple lines) or a control CD4 CAR that lacked the CD3ζ signaling chain (C46CD4CARΔzeta, orange and red dashed lines). (A) Total white blood cell, (B) Platelet, (C), Neutrophil, and (D) Lymphocyte values were measured by automated differential count. Dotted lines represent normal values.
(PDF)

S3 Fig C46CD4CAR cells expand in response to SHIV antigen *in vivo*. (A) % of huCD4+ cells among T cells and (B) Absolute number of huCD4+T cells per ul of peripheral blood. Closed circles and open circles indicate beginning and end of cART, respectively.
(PDF)

S4 Fig HSPC-derived CAR^+^ cells possess specific killing activity *ex vivo*. (A) Study schematic indicating time points from which PBMCs were collected for *ex vivo* killing assay: unsuppressed primary infection (white arrow) and following withdrawal of cART (gray arrow). (B-C) PBMCs from CAR and control animals collected during primary SHIV infection (B) or after cART withdrawal (C) were coincubated for 10 hours with U1 target cells either stimulated to express HIV envelope (Env+) or unstimulated (Env-). (D) Summary of specific killing of mediated by PBMCs from transplanted NHPs. Specific killing is calculated as % killing of target—% killing of control cells.
(PDF)

S5 Fig Natural anti-SHIV T cell response responses in transplanted animals following SHIV challenge and after cART withdrawal. (A) Study schematic indicating time points from which PBMCs were collected for *ex vivo* cytokine assay: unsuppressed primary infection (white arrow) and following withdrawal of cART (gray arrow). Cryopreserved PBMCs were thawed from CAR and control animals during untreated SHIV infection (B) and after cART withdrawal (C) were stimulated with SIVmac peptide pool overnight. Expression of intracellular IFNγ was measured after 6 hours of additional GolgiPlug treatment.
(PDF)

S6 Fig Virus-specific antibody responses in transplanted animals following SHIV challenge. At the indicated time points following SHIV challenge, serum samples were collected from CAR (solid lines) and control animals (dashed lines). ELISA was used to quantify the titer of antibodies directed against whole virus SIVmac239 (A) and HIV-1 SF162 gp120 (B). Titers are calculated as the reciprocal of the highest serum dilution that resulted in an optical density reading greater than the average values obtained with negative control sera plus three standard deviations. Closed circles and open circles indicate beginning and end of cART, respectively.
(PDF)

S7 Fig Plasma pro-inflammatory cytokine measurement for control and CAR NHPs prior to SHIV infection, during untreated SHIV infection, cART treatment and after cART withdrawal. (A) Study schematic indicating time points from which plasma were collected for multiplex cytokine assay. (B) 50ul plasma from CAR and control animals were used in NHP multiplex assay for detection of pro-inflammatory cytokines. Cytokines that has level lower than 2.44pg/ml were marked as N.D (non-detectable). (C) Summary of plasma MCP-1 level in control and CAR animals. (D) Summary of sCD40L level in control and CAR animals.
(PDF)

S8 Fig Surface phenotyping of CAR-expressing cells in tissues. At necropsy, the indicated tissues were collected and measured by flow cytometry. (A) % of huCD4+ cells among T cells in lymphoid tissues and gut. The percentage of huCD4+ cells that were CD4^+^ or CD8^+^ T cells, CD20^+^ B cells, CD14^+^ macrophages/monocytes, and CD2^+^NKG2a^+^ NK cells among control (B-C) or CAR animals (D-E).
(PDF)

S9 Fig CAR animals showed protection of CD4 T cells in the GI tract. (A) CD4%, (B) CD4/8 ratio and (C) CD4 TEM% among CAR and control animals prior to SHIV infection, during primary SHIV infection, during cART treatment and after cART withdrawal. *Data point not available for control 2, CAR 1 and CAR 2 animals.
(PDF)

S10 Fig Normalized SHIV RNA copies from multiple tissues collected at necropsy. Individual values are shown for the indicated CAR and control animals at the indicated tissue sites.
(PDF)
