# Correction: Long-term persistence and function of hematopoietic stem cell-derived chimeric antigen receptor T cells in a nonhuman primate model of HIV/AIDS

**DOI:** 10.1371/journal.ppat.1006891

**Published:** 2018-03-12

**Authors:** Anjie Zhen, Christopher W. Peterson, Mayra A. Carrillo, Sowmya Somashekar Reddy, Cindy S. Youn, Brianna B. Lam, Nelson Y. Chang, Heather A. Martin, Jonathan W. Rick, Jennifer Kim, Nick C. Neel, Valerie K. Rezek, Masakazu Kamata, Irvin S. Y. Chen, Jerome A. Zack, Hans-Peter Kiem, Scott G. Kitchen

The following information is missing from the Funding section: This study was supported by NIH, Office of Research Infrastructure Programs (ORIP) P510D010425 to WaNPRC.
