# Supplementary material for: Long-term persistence and function of hematopoietic stem cell-derived chimeric antigen receptor T cells in a nonhuman primate model of HIV/AIDS
Source: PLoS Pathog. 2017 Dec 28;13(12):e1006753. doi: 10.1371/journal.ppat.1006753 (PMC5746250; doi:10.1371/journal.ppat.1006753)
Supplement: S1 Fig — Four male juvenile pigtail macaques were transplanted with autologous HSPCs transduced with lentiviruses expressing C46CD4CAR (CAR) or C46CD4CARΔZeta (Control). Colony forming assays were plated from a small aliquot of transduced CD34+ cells that were infused into each autologous recipient. “Percent Lenti+ Colonies” represents the number of lentivirus-positive colonies divided by actin-positive colonies, measured by PCR; numerical values are displayed over each bar. (PDF) [file ppat.1006753.s001.pdf]

Supplementary Figures

Supplementary Figure 1

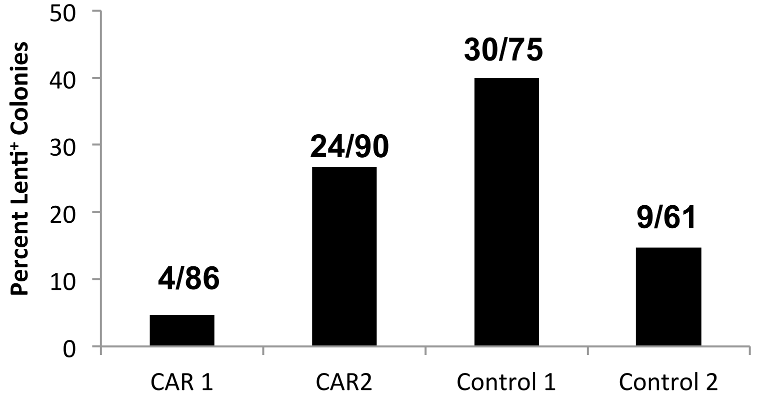

Supplementary Figure 1. Lentiviral gene marking in transduced HSPC infusion products. Four male juvenile pigtailed macaques were transplanted with autologous HSPCs transduced with lentiviruses expressing C46CD4CAR (CAR) or C46CD4CARΔZeta (Control). Colony forming assays were plated from a small aliquot of transduced $CD34^+$ cells that were infused into each autologous recipient. “Percent Lenti+ Colonies” represents the number of lentivirus-positive colonies divided by actin-positive colonies, measured by PCR; numerical values are displayed over each bar.
