# Supplementary material for: Long-term persistence and function of hematopoietic stem cell-derived chimeric antigen receptor T cells in a nonhuman primate model of HIV/AIDS
Source: PLoS Pathog. 2017 Dec 28;13(12):e1006753. doi: 10.1371/journal.ppat.1006753 (PMC5746250; doi:10.1371/journal.ppat.1006753)
Supplement: S2 Fig — Four male juvenile pigtail macaques were transplanted with autologous HSPCs transduced with lentiviruses expressing either CD4 chimeric antigen receptor (C46CD4CAR, green and purple lines) or a control CD4 CAR that lacked the CD3ζ signaling chain (C46CD4CARΔzeta, orange and red dashed lines). (A) Total white blood cell, (B) Platelet, (C), Neutrophil, and (D) Lymphocyte values were measured by automated differential count. Dotted lines represent normal values. (PDF) [file ppat.1006753.s002.pdf]

Supplementary Figure 2

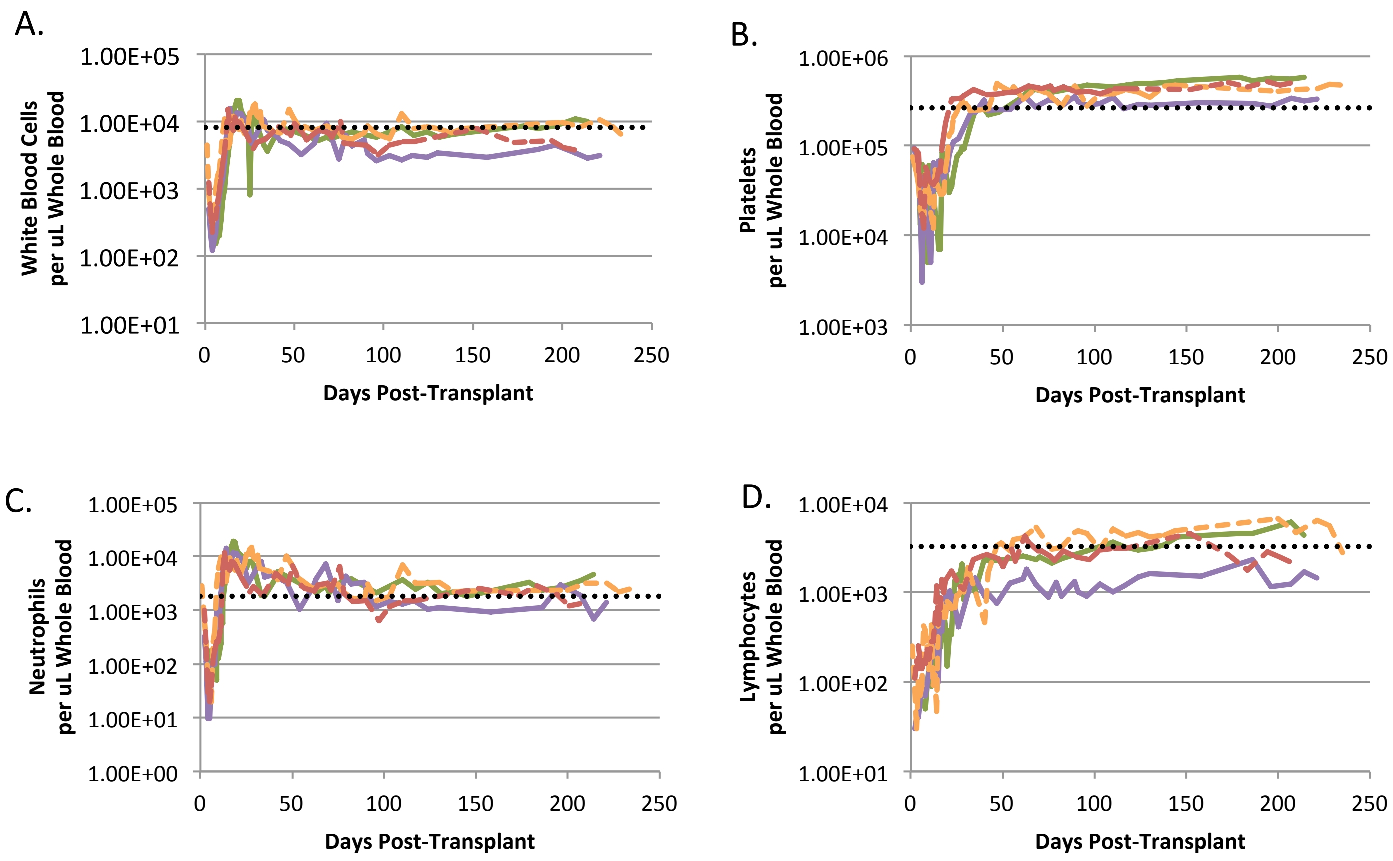

Supplementary Figure 2. Hematopoietic Recovery Following CD4 CAR-HSPC Transplant. Four male juvenile pigtailed macaques were transplanted with autologous HSPCs transduced with lentiviruses expressing either CD4 chimeric antigen receptor (C46CD4CAR, green and purple lines) or a control CD4 CAR that lacked the CD3ζ signaling chain (C46CD4CARΔzeta, orange and red dashed lines). **(A)** Total white blood cell, **(B)** Platelet, **(C)**, Neutrophil, and **(D)** Lymphocyte values were measured by automated differential count. Dotted lines represent normal values.
