# Supplementary material for: Long-term persistence and function of hematopoietic stem cell-derived chimeric antigen receptor T cells in a nonhuman primate model of HIV/AIDS
Source: PLoS Pathog. 2017 Dec 28;13(12):e1006753. doi: 10.1371/journal.ppat.1006753 (PMC5746250; doi:10.1371/journal.ppat.1006753)
Supplement: S3 Fig — (A) % of huCD4+ cells among T cells and (B) Absolute number of huCD4+T cells per ul of peripheral blood. Closed circles and open circles indicate beginning and end of cART, respectively. (PDF) [file ppat.1006753.s003.pdf]

Supplementary Figure 3

A.

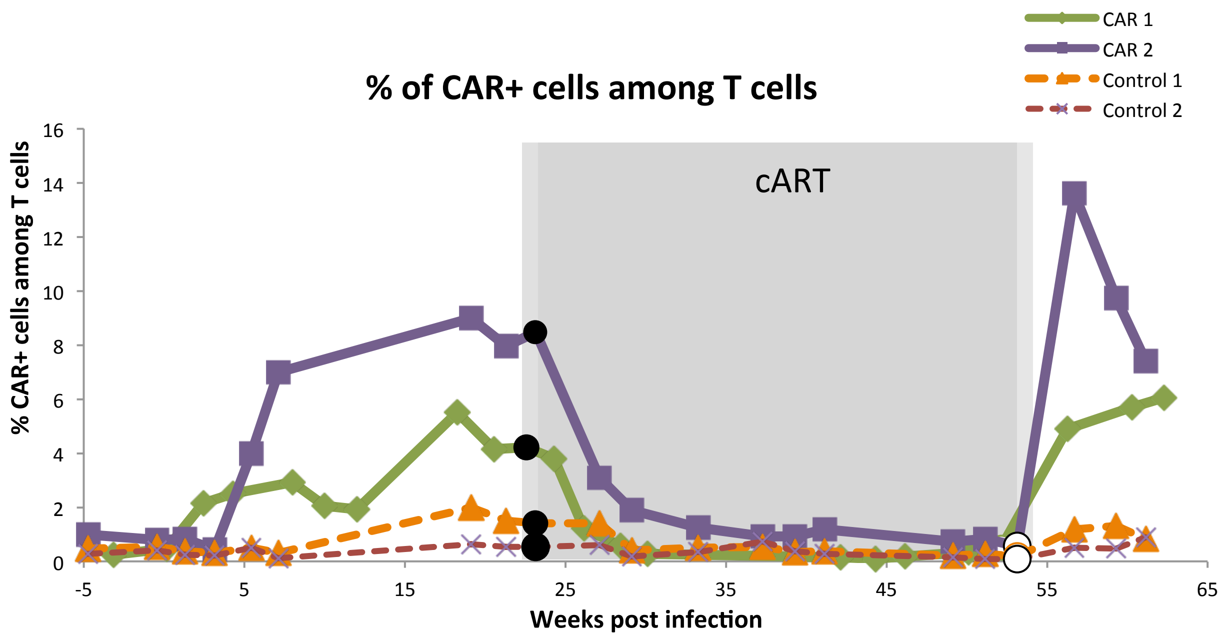

B.

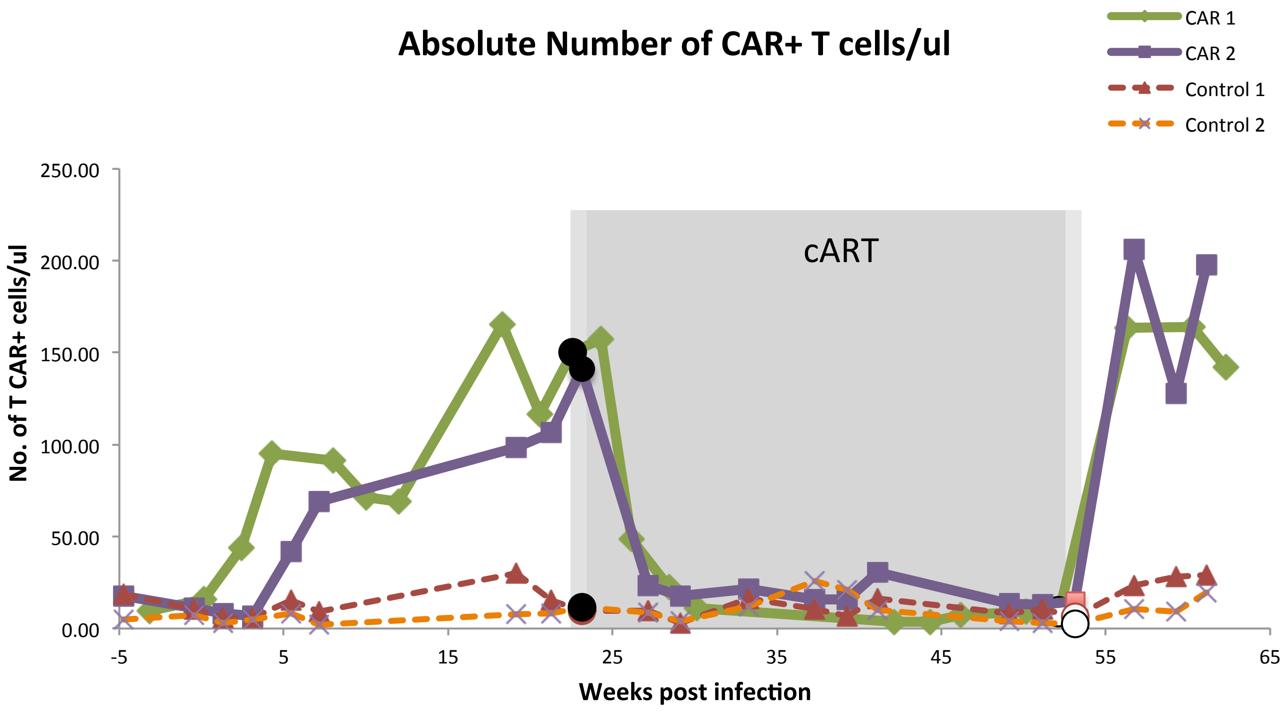

Supplementary Figure 3: C46CD4CAR cells expand in response to SHIV antigen *in vivo.* (A) % of huCD4+ cells among T cells and **(B)** Absolute number of huCD4+T cells per ul of peripheral blood. Closed circles and open circles indicate beginning and end of cART, respectively.
