# Supplementary material for: Long-term persistence and function of hematopoietic stem cell-derived chimeric antigen receptor T cells in a nonhuman primate model of HIV/AIDS
Source: PLoS Pathog. 2017 Dec 28;13(12):e1006753. doi: 10.1371/journal.ppat.1006753 (PMC5746250; doi:10.1371/journal.ppat.1006753)
Supplement: S4 Fig — (A) Study schematic indicating time points from which PBMCs were collected for ex vivo killing assay: unsuppressed primary infection (white arrow) and following withdrawal of cART (gray arrow). (B-C) PBMCs from CAR and control animals collected during primary SHIV infection (B) or after cART withdrawal (C) were coincubated for 10 hours with U1 target cells either stimulated to express HIV envelope (Env+) or unstimulated (Env-). (D) Summary of specific killing of mediated by PBMCs from transplanted NHPs. Specific killing is calculated as % killing of target—% killing of control cells. (PDF) [file ppat.1006753.s004.pdf]

Supplementary Figure 4

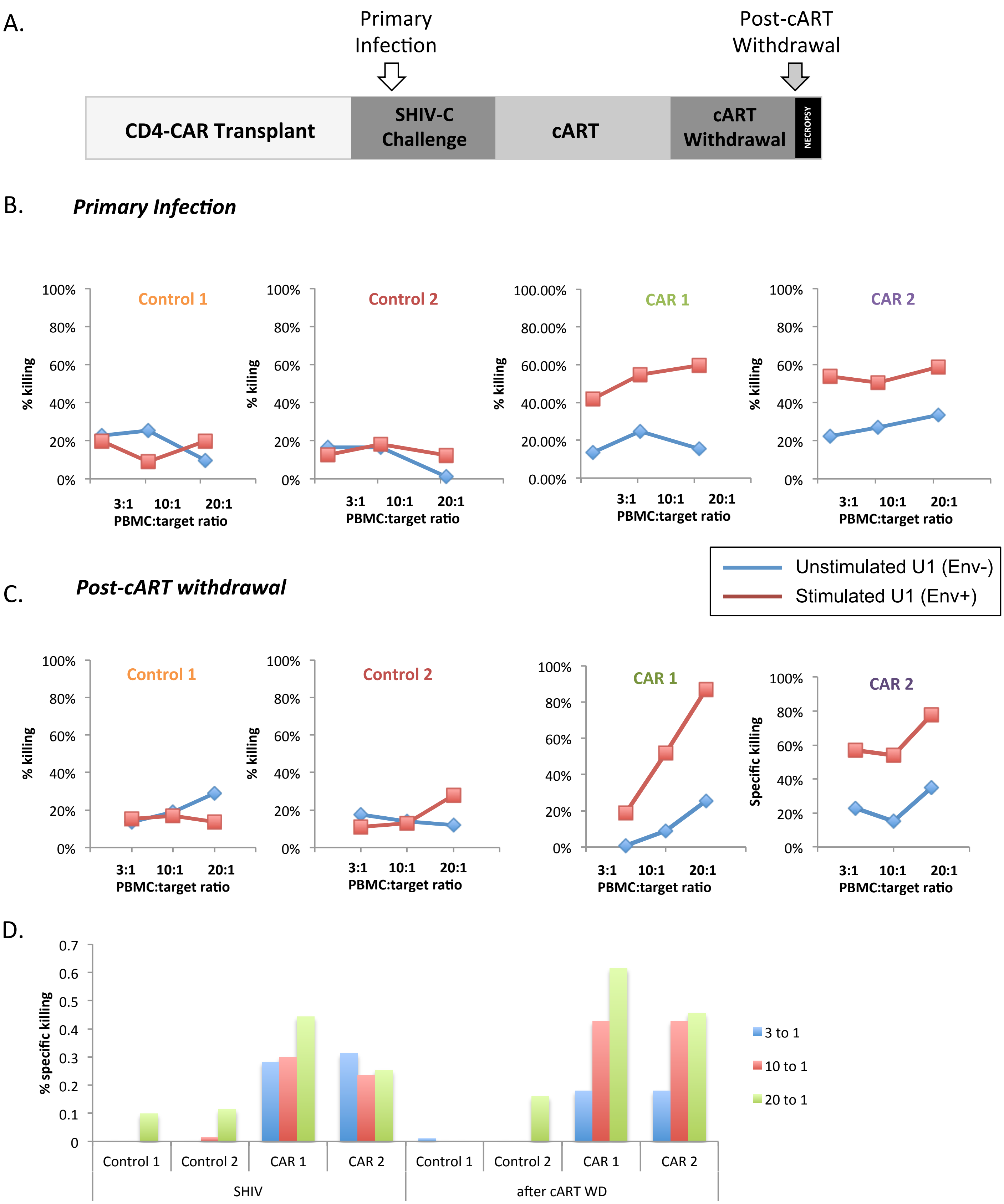

Supplementary Figure 4: HSPC-Derived CAR+ Cells Possess Specific Killing Activity *ex vivo*. **(A)** Study schematic indicating time points from which PBMCs were collected for *ex vivo* killing assay: unsuppressed primary infection (white arrow) and following withdrawal of cART (gray arrow). **(B-C)** PBMCs from CAR and control animals collected during primary SHIV infection (B) or after cART withdrawal (C) were coincubated for 10 hours with U1 target cells either stimulated to express HIV envelope (Env+) or unstimulated (Env-). **(D)** Summary of specific killing of mediated by PBMCs from transplanted NHPs. Specific killing is caculated as % killing of target - % killing of control cells.
