# Supplementary material for: Long-term persistence and function of hematopoietic stem cell-derived chimeric antigen receptor T cells in a nonhuman primate model of HIV/AIDS
Source: PLoS Pathog. 2017 Dec 28;13(12):e1006753. doi: 10.1371/journal.ppat.1006753 (PMC5746250; doi:10.1371/journal.ppat.1006753)
Supplement: S5 Fig — (A) Study schematic indicating time points from which PBMCs were collected for ex vivo cytokine assay: unsuppressed primary infection (white arrow) and following withdrawal of cART (gray arrow). Cryopreserved PBMCs were thawed from CAR and control animals during untreated SHIV infection (B) and after cART withdrawal (C) were stimulated with SIVmac peptide pool overnight. Expression of intracellular IFNγ was measured after 6 hours of additional GolgiPlug treatment. (PDF) [file ppat.1006753.s005.pdf]

Supplementary Figure 5

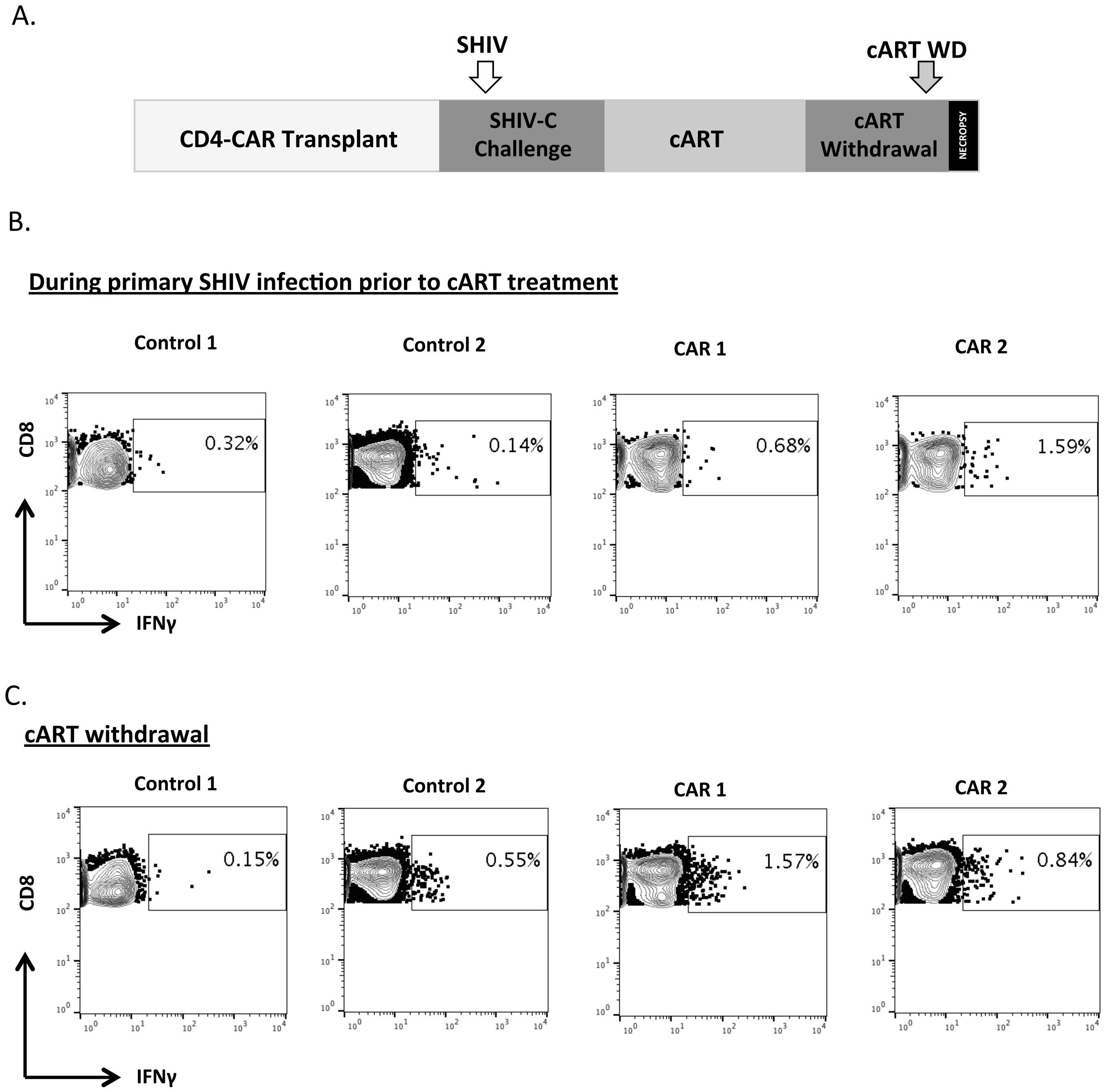

Supplementary Figure 5: Natural anti-SHIV T cell response Responses in Transplanted Animals Following SHIV Challenge and after cART withdrawal. (A) Study schematic indicating time points from which PBMCs were collected for *ex vivo* cytokine assay: unsuppressed primary infection (white arrow) and following withdrawal of cART (gray arrow). Cryopreserved PBMCs were thawed from CAR and control animals during untreated SHIV infection **(B)** and after cART withdrawal **(C)** were stimulated with SIVmac peptide pool overnight. Expression of intracellular IFNγ was measured after 6 hours of additional GolgiPlug treatment.
