# Supplementary material for: Long-term persistence and function of hematopoietic stem cell-derived chimeric antigen receptor T cells in a nonhuman primate model of HIV/AIDS
Source: PLoS Pathog. 2017 Dec 28;13(12):e1006753. doi: 10.1371/journal.ppat.1006753 (PMC5746250; doi:10.1371/journal.ppat.1006753)
Supplement: S6 Fig — At the indicated time points following SHIV challenge, serum samples were collected from CAR (solid lines) and control animals (dashed lines). ELISA was used to quantify the titer of antibodies directed against whole virus SIVmac239 (A) and HIV-1 SF162 gp120 (B). Titers are calculated as the reciprocal of the highest serum dilution that resulted in an optical density reading greater than the average values obtained with negative control sera plus three standard deviations. Closed circles and open circles indicate beginning and end of cART, respectively. (PDF) [file ppat.1006753.s006.pdf]

Supplementary Figure 6

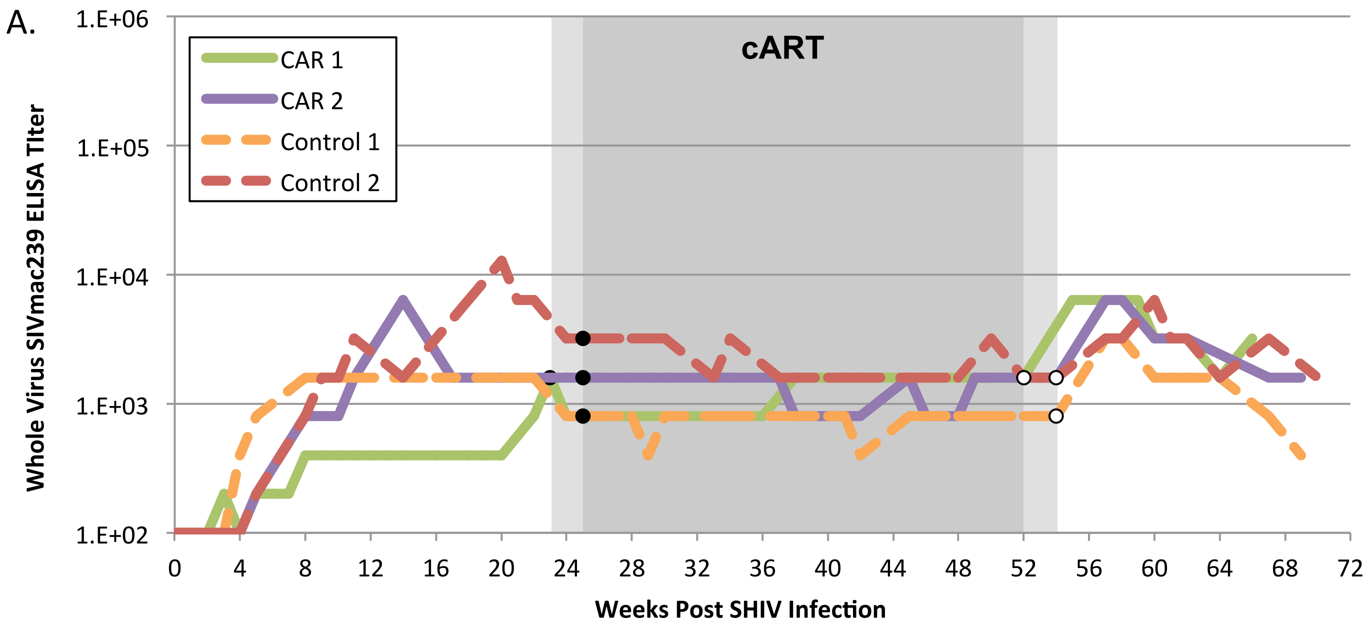

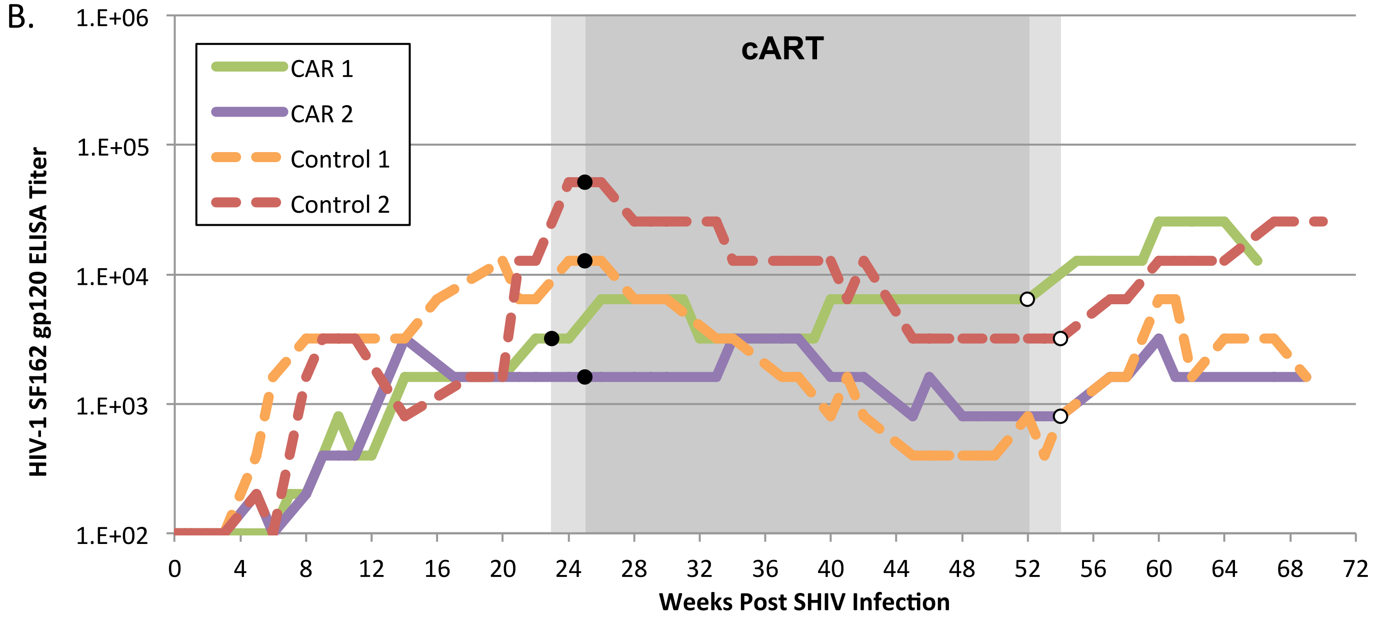

Supplementary Figure 6: Virus-specific antibody responses in transplanted animals following SHIV challenge. At the indicated time points following SHIV challenge, serum samples were collected from CAR (solid lines) and control animals (dashed lines). ELISA was used to quantify the titer of antibodies directed against whole virus SIVmac239 (A) and HIV-1 SF162 gp120 (B). Titers are calculated as the reciprocal of the highest serum dilution that resulted in an optical density reading greater than the average values obtained with negative control sera plus three standard deviations. Closed circles and open circles indicate beginning and end of cART, respectively.
