# Supplementary material for: Long-term persistence and function of hematopoietic stem cell-derived chimeric antigen receptor T cells in a nonhuman primate model of HIV/AIDS
Source: PLoS Pathog. 2017 Dec 28;13(12):e1006753. doi: 10.1371/journal.ppat.1006753 (PMC5746250; doi:10.1371/journal.ppat.1006753)
Supplement: S7 Fig — (A) Study schematic indicating time points from which plasma were collected for multiplex cytokine assay. (B) 50ul plasma from CAR and control animals were used in NHP multiplex assay for detection of pro-inflammatory cytokines. Cytokines that has level lower than 2.44pg/ml were marked as N.D (non-detectable). (C) Summary of plasma MCP-1 level in control and CAR animals. (D) Summary of sCD40L level in control and CAR animals. (PDF) [file ppat.1006753.s007.pdf]

Supplementary Figure 7

A.

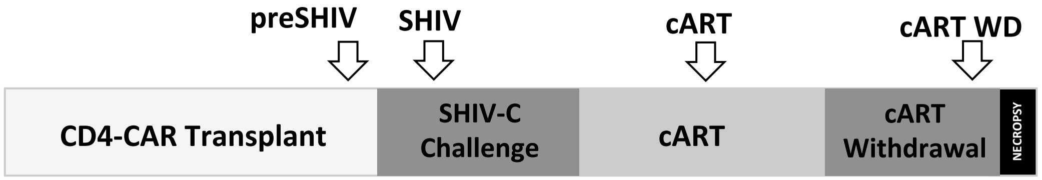

B.

		Cytokines (*pg/ml) Detection range: 2.44-10,000g/ml								
		**IFNγ**	**IL-1β**	**IL-2**	**IL-6**	**MCP-1**	**MIP1β**	**MIP1α**	**sCD40L**	**TNFα**
**CAR 1**	PreSHIV	N.D	N.D	N.D	N.D	377.6	46.88	N.D	1059.0	14.7
	SHIV	N.D	N.D	N.D	N.D	556.3	33.88	N.D	287.9	4.6
	cART	N.D	N.D	N.D	N.D	533.8	38.06	N.D	117.9	9.8
	cART WD	N.D	N.D	N.D	N.D	306.1	40.83	N.D	3.9	N.D
**CAR 2**	PreSHIV	N.D	N.D	N.D	N.D	376.5	N.D	N.D	45710.5	12.2
	SHIV	N.D	N.D	9.1	N.D	213.3	N.D	N.D	96.0	21.7
	cART	N.D	N.D	N.D	N.D	277.6	N.D	N.D	331.0	N.D
	cART WD	N.D	N.D	49.2	N.D	357.9	53.155	23.31	2971.6	49.82
**Control 1**	PreSHIV	N.D	N.D	N.D	N.D	259.5	N.D	N.D	104060.2	32.93
	SHIV	N.D	N.D	N.D	N.D	288.8	N.D	N.D	918676.7	19.39
	cART	N.D	N.D	N.D	N.D	254.0	N.D	N.D	76.2	N.D
	cART WD	N.D	N.D	N.D	N.D	255.8	N.D	N.D	24390.0	14.67
**Control 2**	PreSHIV	N.D	N.D	9.1	N.D	359.7	73.08	N.D	23705.2	9.78
	SHIV	N.D	N.D	50.6	N.D	227.2	38.185	26.91	7523.9	70.73
	cART	N.D	N.D	37.1	N.D	234.8	46.72	N.D	280.5	39.46
	cART WD	N.D	N.D	N.D	N.D	320.0	N.D	N.D	115807.0	12.23

C.

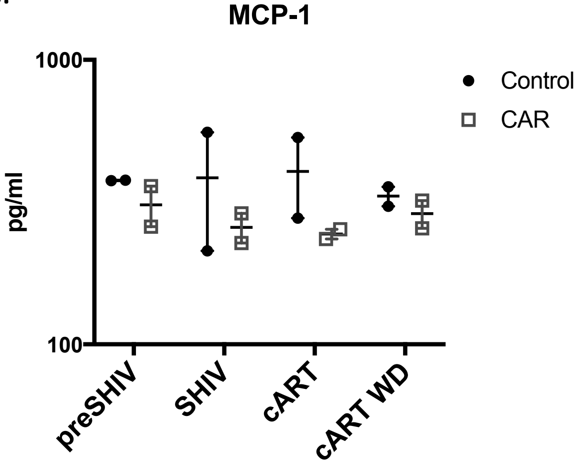

D.

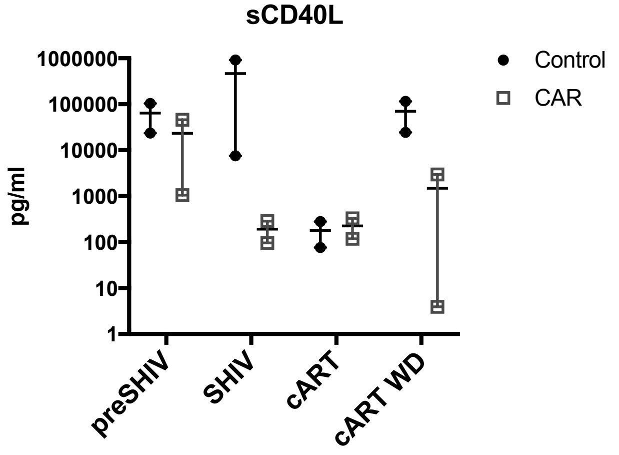

Supplementary Figure 7: Plasma pro-inflammatory cytokine measurement for control and CAR NHPs prior to SHIV infection, during untreated SHIV infection, cART treatment and after cART withdrawal. (A) Study schematic indicating time points from which plasma were collected for multiplex cytokine assay. (**B)** 50ul plasma from CAR and control animals were used in NHP multiplex assay for detection of pro-inflammatory cytokines. Cytokines that has level lower than 2.44pg/ml were marked as N.D (non-detectable). **(C)** Summary of plasma MCP-1 level in control and CAR animals. **(D)** Summary of sCD40L level in control and CAR animals.
