# Supplementary material for: Long-term persistence and function of hematopoietic stem cell-derived chimeric antigen receptor T cells in a nonhuman primate model of HIV/AIDS
Source: PLoS Pathog. 2017 Dec 28;13(12):e1006753. doi: 10.1371/journal.ppat.1006753 (PMC5746250; doi:10.1371/journal.ppat.1006753)
Supplement: S8 Fig — At necropsy, the indicated tissues were collected and measured by flow cytometry. (A) % of huCD4+ cells among T cells in lymphoid tissues and gut. The percentage of huCD4+ cells that were CD4+ or CD8+ T cells, CD20+ B cells, CD14+ macrophages/monocytes, and CD2+NKG2a+ NK cells among control (B-C) or CAR animals (D-E). (PDF) [file ppat.1006753.s008.pdf]

Supplementary Figure 8

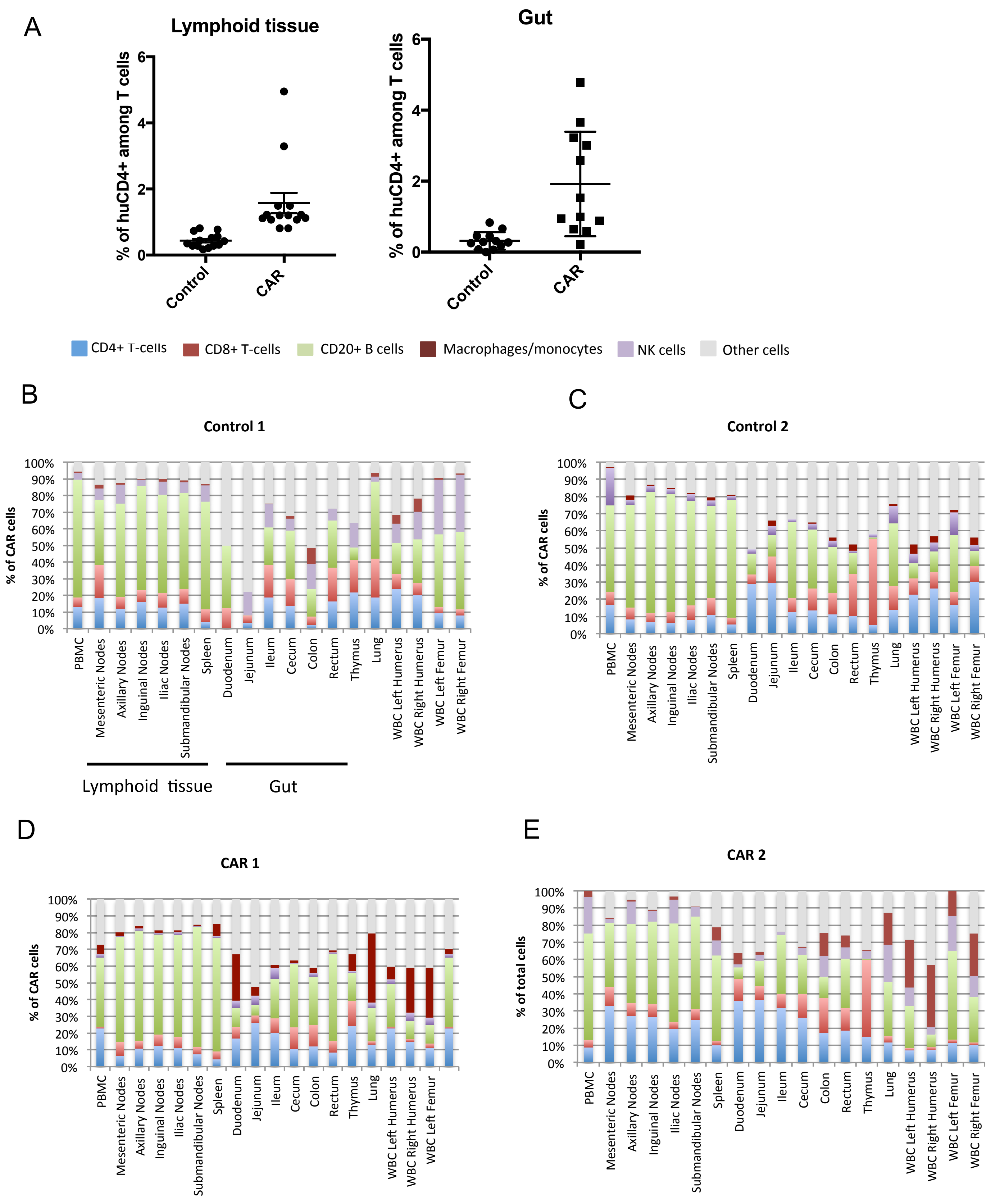

Supplementary Figure 8: Surface phenotyping of CAR-expressing cells in tissues. At necropsy, the indicated tissues were collected and measured by flow cytometry. **(A)** % of huCD4+ cells among T cells in lymphoid tissues and gut. The percentage of huCD4+ cells that were CD4^{+} or CD8^{+} T cells, CD20^{+} B cells, CD14^{+} macrophages/monocytes, and CD2^{+}NKG2a^{+} NK cells among control (B-C) or CAR animals (D-E).
