# Supplementary material for: Long-term persistence and function of hematopoietic stem cell-derived chimeric antigen receptor T cells in a nonhuman primate model of HIV/AIDS
Source: PLoS Pathog. 2017 Dec 28;13(12):e1006753. doi: 10.1371/journal.ppat.1006753 (PMC5746250; doi:10.1371/journal.ppat.1006753)
Supplement: S9 Fig — (A) CD4%, (B) CD4/8 ratio and (C) CD4 TEM% among CAR and control animals prior to SHIV infection, during primary SHIV infection, during cART treatment and after cART withdrawal. *Data point not available for control 2, CAR 1 and CAR 2 animals. (PDF) [file ppat.1006753.s009.pdf]

# Supplementary Figure 9

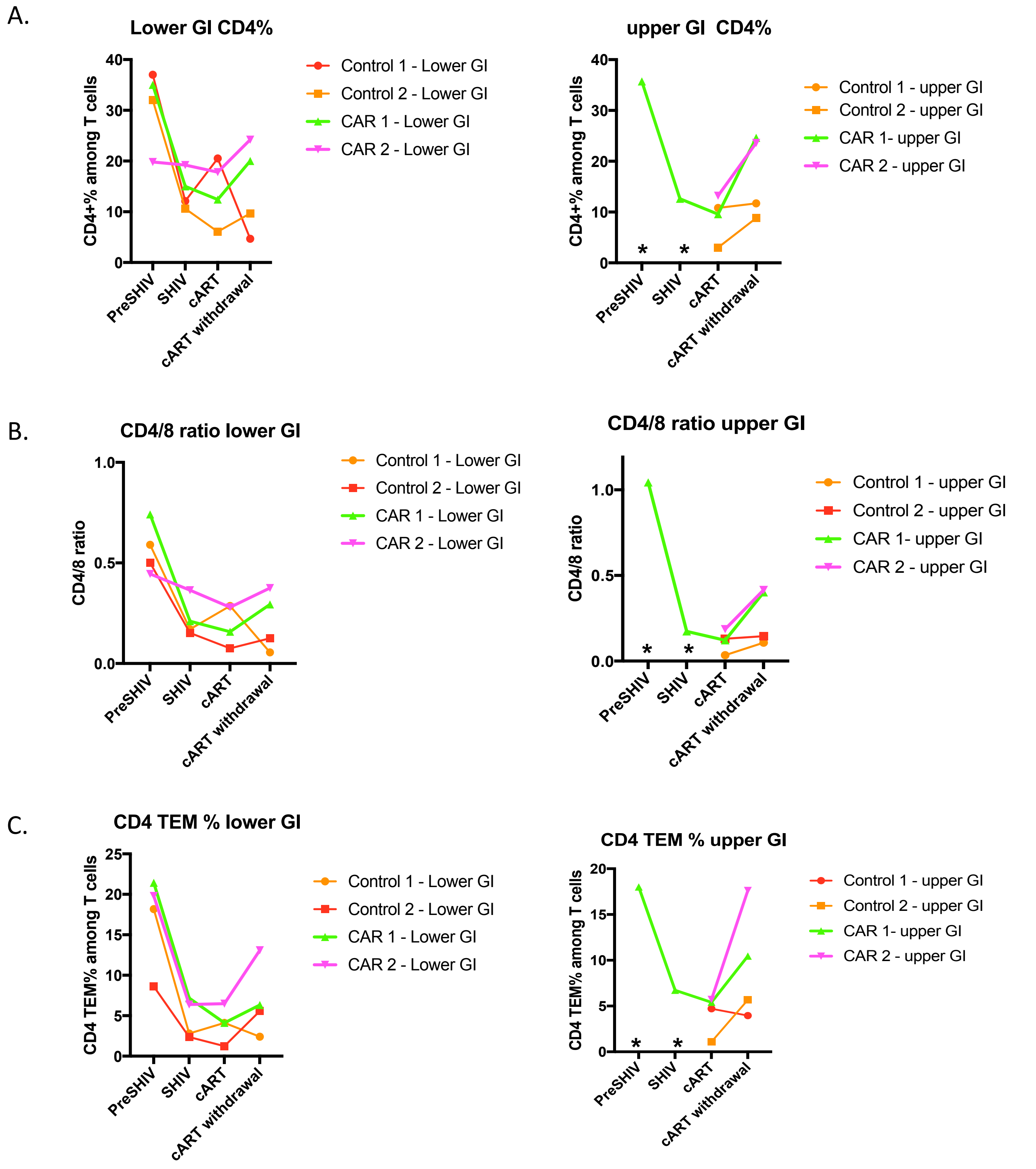


**Supplementary Figure 9: CAR animals showed protection of CD4 T cells in the GI tract. (A)** CD4%, (**B**) CD4/8 ratio and **(C)** CD4 TEM% among CAR and control animals prior to SHIV infection, during primary SHIV infection, during cART treatment and after cART withdrawal. *Data point not available for control 2, CAR 1 and CAR 2 animals.
