# Supplementary material for: Long-term persistence and function of hematopoietic stem cell-derived chimeric antigen receptor T cells in a nonhuman primate model of HIV/AIDS
Source: PLoS Pathog. 2017 Dec 28;13(12):e1006753. doi: 10.1371/journal.ppat.1006753 (PMC5746250; doi:10.1371/journal.ppat.1006753)
Supplement: S10 Fig — Individual values are shown for the indicated CAR and control animals at the indicated tissue sites. (PDF) [file ppat.1006753.s010.pdf]

Supplementary Figure 10

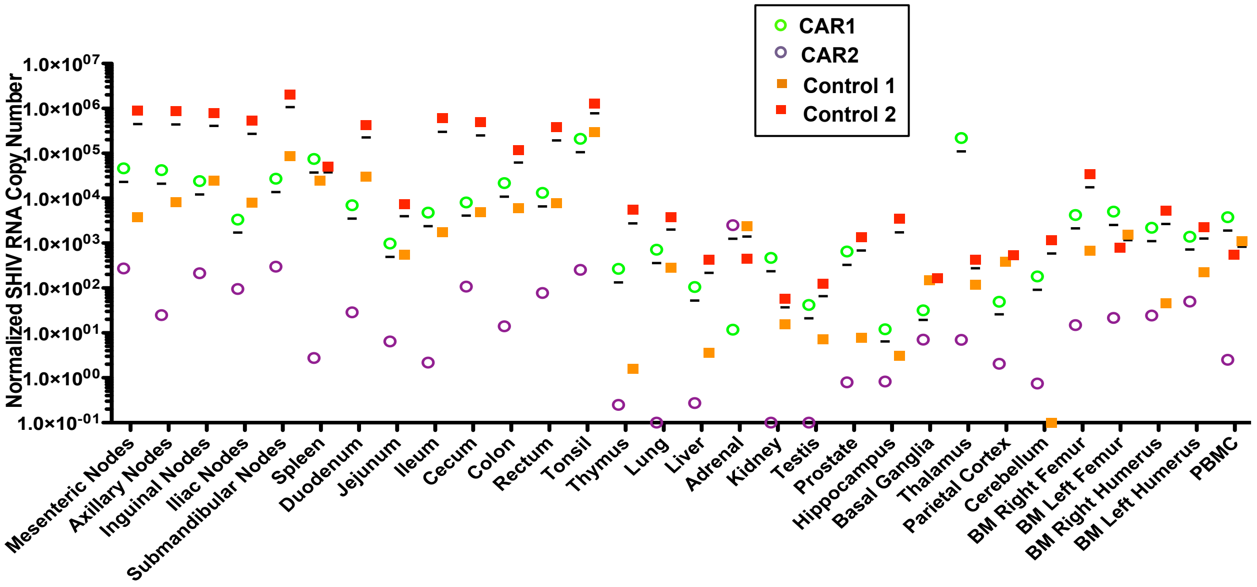

Supplementary Figure 10: Normalized SHIV RNA copies from multiple tissues collected at necropsy. Individual values are shown for the indicated CAR and control animals at the indicated tissue sites.
